# Reproduction and In-Depth Evaluation of Genome-Wide Association Studies and Genome-Wide Meta-analyses Using Summary Statistics

**DOI:** 10.1534/g3.116.038877

**Published:** 2017-01-24

**Authors:** Yao-Fang Niu, Chengyin Ye, Ji He, Fang Han, Long-Biao Guo, Hou-Feng Zheng, Guo-Bo Chen

**Affiliations:** * State Key Laboratory of Rice Biology, China National Rice Research Institute, Hangzhou, 310006 Zhejiang, China; † Department of Health Management, College of Medicine, Hangzhou Normal University, Hangzhou, 310021 Zhejiang, China; ‡ Department of Neurology, Peking University Third Hospital, Beijing 100191, China; § Department of Pulmonary and Critical Care Medicine, Peking University People’s Hospital, Beijing 100044, China; ** Institute of Aging Research, College of Medicine, Hangzhou Normal University, Hangzhou, 310021 Zhejiang, China; †† The Affiliated Hospital of Hangzhou Normal University, Hangzhou, 310015 Zhejiang, China; ‡‡ Evergreen Landscape and Architecture Studio, Hangzhou, 310026 Zhejiang, China

**Keywords:** GWAS, *Arabidopsis*, magnesium, transparency, reproducibility, naïve summary statistics, meta-analyses, GWAMA, GEAR

## Abstract

In line with open-source genetics, we report a novel linear regression technique for genome-wide association studies (GWAS), called Open GWAS algoriTHm (OATH). When individual-level data are not available, OATH can not only completely reproduce reported results from an experimental model, but also recover underreported results from other alternative models with a different combination of nuisance parameters using naïve summary statistics (NSS). OATH can also reliably evaluate all reported results in-depth (*e.g.*, *p*-value variance analysis), as demonstrated for 42 *Arabidopsis* phenotypes under three magnesium (Mg) conditions. In addition, OATH can be used for consortium-driven genome-wide association meta-analyses (GWAMA), and can greatly improve the flexibility of GWAMA. A prototype of OATH is available in the Genetic Analysis Repository (https://github.com/gc5k/GEAR).

Reproducibility and transparency are the cornerstones of scientific integrity. In addition to artifacts that may compromise a study, analysis itself is becoming more complicated and poses another obstacle to reproducing discoveries. For big data studies involving high-throughput computation, such as GWAS, the reported findings are subject to criticism, as the results may differ among models even when the experimental design is sound. Therefore, the choice of a model as well as its conclusion (*i.e.*, false positive or false negative) are often justified by an analyst’s prior knowledge (Aschard *et al.* 2015; Day *et al.* 2016). However, given practical constraints, such as data sharing policies and computational burden, it is not feasible to present all possible results found under alternative models. Although many consortia encourage open-source genetics and have released GWAS summary statistics, including the Genetic Investigation of Anthropometric Traits (GIANT) Consortium and the Psychiatric Genomic Consortia (PGC), it is still difficult to thoroughly evaluate a published study. Consequently, reproducibility and the success rate of subsequent studies are hampered. What kind of method and set of summary statistics are needed to fully reproduce results and to explore studies using unreported analyses?

Statistical analyses can be reproduced in the absence of individual-level data; this is possible due to the theory of sufficient statistics (Fisher 1921). In this study, we propose a complementary method to reproduce each GWAS hit in the absence of shared original data. We report an algorithm called OATH that works directly on summary statistics. When individual-level data are not available, OATH can not only completely reproduce the reported results from an experimental model but also recover underreported results from other alternative models using only summary statistics. The utility of OATH will be demonstrated for 42 phenotypes: 14 traits of 295 *Arabidopsis* inbred lines grown under three Mg conditions.

Furthermore, as OATH is based on linear regression, its application to other analyses is possible as long as linear regression was employed. For example, OATH can be embedded into consortium-driven GWAMA. Without loss of generality, the literature-driven meta-analyses can be considered a “retrospective” study, which is often an irreversible process under which a meta-analyses conductor can rarely customize the summary statistics. In contrast, a consortium-driven GWAMA can be a “prospective” study; quality control can be conducted more thoroughly (Chen *et al.* 2017) and the summary statistics from each cohort can be customized under the request of the consortium. As demonstrated below with two Chinese GWAS cohorts, a consortium-driven GWAMA can more efficiently adjust covariates using OATH.

## Materials and Methods

We begin this section with a brief explanation of the OATH algorithm; a more detailed description can be found in the Supplemental Material. To demonstrate the use of OATH, an introduction of *Arabidopsis* GWAS data under Mg treatments and two Chinese GWAS cohorts will follow.

### OATH

For a saturated GWAS analysis, its multiple regression model is written as (for the ease of discussion, all variables are centered, but the method can be applied to data not centered)

$$y=X_{i}\beta_{i}+e,$$ (1)

in which y is the observed phenotype of n individuals, $X_{i}=\left[x_{i}^{*}:z_{1}:\ldots :z_{m}\right],$ and e is the residual. $x_{i}$ codes the counts of the reference alleles at the ith locus and $z_{j}$ is the jth covariate. $\beta_{i}^{\prime }=\left[\beta_{i}^{*},\beta_{1},\beta_{2},\ldots ,\beta_{m}\right],$ in which $\beta_{i}^{*}$ is the effect size of the marker and $\beta_{j}$ is the partial regression coefficient. The least-squares estimator is $\beta_{i}=\Omega_{i}^{-1}X_{i}y\;,$ in which $\Omega_{i}=X_{i}^{\prime }X_{i}.$ Both $X_{i}$ and y are individual-level data in the estimator.

The least-squares estimator for β can also be expressed in the following form (see Supplemental Material; hereafter referred to as OATH):

$$\hat{\beta_{i}}=\Omega_{i}^{-1}\Lambda_{i}\hat{b_{i}},$$ (2)

in which $\Lambda_{i}$ is the diagonal of $\Omega_{i}.$ $\hat{b_{i}^{\prime }}=\left[\hat{b_{i}^{*}},\hat{b_{1}},\hat{b_{2}},\ldots ,\hat{b_{m}}\right],$ in which $\hat{b_{i}}=\sigma_{y,z_{i}}/\sigma_{z_{i}}^{2}$ is for $y=z_{i}b_{i}+\varepsilon .$ The variance–covariance matrix of $\hat{\beta_{i}}$ is

$$\hat{\sigma_{\beta_{i}}^{2}}=\left(\frac{\sigma_{y}^{2}-\hat{\beta_{i}^{\prime }}\Lambda_{i}\hat{b_{i}}}{n-m-1}\right)\Omega_{i}^{-1}.$$ (3)

**Figure 1:**
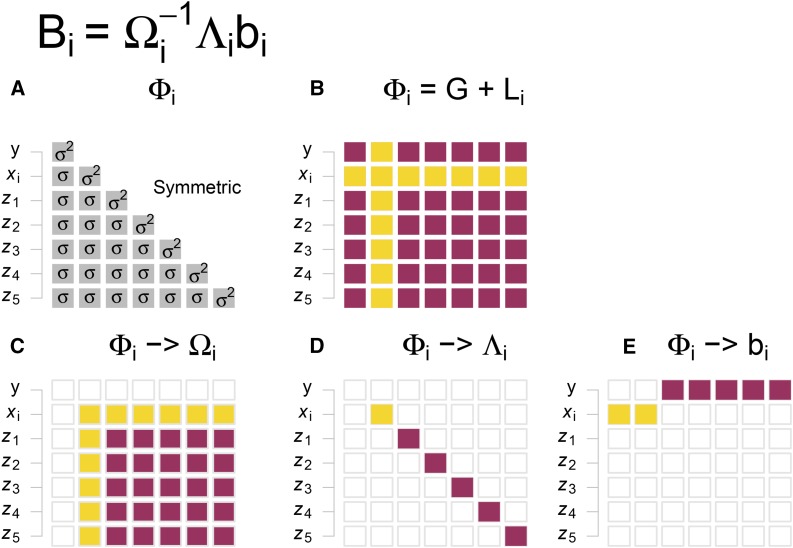
Schematic illustration of open GWAS algorithm (OATH). The first row is the OATH equation. The second row shows (A) the sufficient statistics $\Phi_{i},$ a symmetric matrix, and (B) $\Phi_{i}$ can be split into generic *G* (red) and locus-specific l (yellow) parts. The third row represents how the elements in $\Phi_{i}$ can be extracted to build (C) $\Omega_{i},$ (D) $\Lambda_{i},$ and (E) $b_{i},$ respectively. GWAS, genome-wide association studies; OATH, Open GWAS algoriTHm.

The information [known as sufficient statistics for data reduction (Fisher 1921)] required for Equations 2 and 3 is contained in $\Phi_{i}=\left(\begin{array}{cc} \begin{array}{cc} \sigma_{y}^{2} & \sigma_{y,x_{i}}\\ \sigma_{x_{i},y} & \sigma_{x_{i}}^{2} \end{array} & \begin{array}{ccc} \sigma_{y,z_{1}} & \ldots & \sigma_{y,z_{m}}\\ \sigma_{x_{i},x_{1}} & \cdots & \sigma_{x_{i},z_{m}} \end{array}\\ \begin{array}{cc} \sigma_{z_{1},y} & \sigma_{z_{1},x_{i}}\\ \vdots & \vdots \\ \sigma_{z_{m},y} & \sigma_{z_{m},x_{i}} \end{array} & \begin{array}{ccc} \sigma_{z_{1}}^{2} & \cdots & \sigma_{z_{1},z_{m}}\\ \vdots & \ddots & \vdots \\ \sigma_{z_{m},x_{1}} & \cdots & \sigma_{z_{m}}^{2} \end{array} \end{array}\right),$ the variance–covariance matrix of all variables in Equation 1; no individual-level data are needed. As illustrated in Figure 1, all elements for Equations 2 and 3 can be extracted from $\Phi_{i}.$ Rather than summary statistics from complicated models, $\Phi_{i}$ involves variance and covariance only; therefore, we call them NSS in the text below. Of note, as the second row/column of $\Phi_{i}$ is locus-specific, only the locus-specific part of $\Phi_{i}$ should be provided for each locus (Figure 1 and Supplemental Material, File S1).

In general, Equation 2 can be written as $\hat{\beta_{i.s}}=\Omega_{i.s}^{-1}\Lambda_{i.s}\hat{b_{i.s}},$ s indicating the set of covariates included. If any covariates are dropped from $X_{i},$ Equations 2 and 3 can be tailored to generate a corresponding estimate for the target marker effect $\hat{\beta_{i}^{*}}.$ Thus, recovering underreported results for any combination of covariates is possible if the summary statistic $\Phi_{i}$ is provided.

One possible application for OATH is GWAMA. If each cohort sends $\Phi_{i}$ to the central hub, the whole GWAMA gains more flexibility because the central hub will be able to customize the GWAS model to any combination of covariates. The technical details on how to integrate OATH into GWAMA can be found in File S1.

### Arabidopsis GWAS data

The seeds of all 295 lines were acquired from the *Arabidopsis* Biological Resources Center stock. Then, 234 accessions were sampled from 1307 worldwide accessions, which were genotyped using a 250 K single nucleotide polymorphism (SNP) chip (Horton *et al.* 2012), and 61 were extracted from the *Arabidopsis* 1001 Genomes Project (http://1001genomes.org) (Figure S1A in File S2). The geographical distribution of the 295 lines was consistent with the *Arabidopsis* lines collected in RegPanel (http://regmap.uchicago.edu) (see Figure S1B in File S2). After quality control [triallelic or tetra-allelic loci, minor allele frequency (MAF) < 0.05, genotyping rate < 0.998, and homozygosity rate < 0.99 were removed], 156,744 biallelic loci remained for 42 GWAS (Figure S2 in File S2). Genetic relatedness was estimated using these 156,744 markers, resulting in a 295×295 genetic relationship matrix (GRM). The eigenvectors were estimated in the GRM.

The 295 inbred lines were grown under three Mg conditions: the low, normal, and high conditions contained 1, 1000, and 10,000 µM MgSO_4_, respectively, which was in accordance with the concentrations of Mg^2+^ in soil solutions (Hariadi and Shabala 2004). Fourteen traits were investigated under each treatment: seven were morphological traits and seven were nutrient concentration traits (Table 1). Under the three treatments, there were 42 total phenotypes for each line (Figure S3 in File S2). To reduce environmental influences, the median value of biological replicates was used as the phenotypic value. To reduce the maternal effects prior to phenotyping, inbred lines were grown for one generation under controlled greenhouse conditions at Zhejiang University (N30°18′25, E120°04′54), Hangzhou, Zhejiang Province, China, in 2015. For the ease of analysis, each phenotype was standardized (Figure S4 and Figure S5 in File S2). See the supplementary notes in File S1 for more details on these traits.

**Table 1:** Fourteen *Arabidopsis* traits investigated under three magnesium (Mg) treatments

Trait Category	Trait Identifier	Full Name of Trait	Unit*^a^*	Trait Description	Annotation
MT	RGT	Days to root germination	d	The number of days from seeding until emergence, with more than half of seedlings having a first radicle	Root germination and lateral root number data were shown as the value obtained in low- or high-Mg treatment minus those under normal-Mg treatment. The primary root values for the low-Mg or high-Mg treatment were then divided by values obtained from normal-Mg treatment
	PRL	Primary root length	cm	After 8 d of growth under the treatments, plants were flattened directly on agar and imaged using a camera	
	LRN	Lateral root number	cm	After 8 d growth of under the treatments, plants were flattened directly on agar and imaged using a camera	
	SGT	Days to shoot germination	d	Normal	Shoot germination data were shown as the value obtained in low- or high-Mg treatment minus those under normal-Mg treatment. The epicotyl length and rosette width values for low-Mg or high-Mg treatment were then divided by values obtained from normal-Mg treatment
	EL	Epicotyl length	cm	After 8 d of growth under the treatments, plants were flattened directly on agar and imaged using a camera	
	RL	Rosette width length	cm	After 8 d of growth under the treatments, plants were flattened directly on agar and imaged using a camera	
	Biomass	Fresh weight of plants	mg	All fully expanded and nonlesioned seedlings were collected from four plants for each accession and weighed to obtain fresh weight measurements. The results represent average values across all available replicates	Biomass and nutrient concentration data were calculated as the ratio of the treatment value (low Mg or high Mg) divided by the normal, in which seeds were germinated in normal Mg
NCT	K	Potassium concentration per plant	mg/g	Elemental analysis was performed with an ICP-MS (Agilent 7500a). All samples were normalized to calculated weights as previously described. The results represent average values across all available replicates	
	Ca	Calcium concentration per plant	mg/g		
	Mg	Magnesium concentration per plant	mg/g		
	S	Sulfur concentration per plant	mg/g		
	Fe	Iron concentration per plant	mg/g		
	Mn	Manganese concentration per plant	mg/g		
	Na	Sodium concentration per plant	mg/g		

MT, morphological traits; NCT nutrition concentration traits; ICP-MS, inductively-coupled plasma mass spectrometry.

a For NCT, the units are measured for fresh weight.

### Two Chinese GWAS cohorts

Two Chinese GWAS cohorts, NA (Han *et al.* 2013) and SLE (Han *et al.* 2009), were used to demonstrate the application of OATH to consortium-driven meta-analyses. The NA cohort was originally recruited for the study of narcolepsy, an autoimmune disorder affecting hypocretin (orexin) neurons; 3191 samples were genotyped. The SLE cohort was recruited for the study of systemic lupus erythematosus in the Chinese population; 2309 samples were genotyped. In order to mimic a consortium-driven GWAMA, these two GWAS cohorts provided the required NSS to the central hub. Using the meta-PCA technique (Chen *et al.* 2017), the general genotyping quality of these two cohorts was validated by the GWAMA central hub, based only on the reported allele frequencies; individual-level data were not required (Figure S6 in File S2).

### Data availability

The authors state that all data necessary for confirming the conclusions presented in the article are represented fully within the article.

## Results

### An OATH simulation example

**Figure 2:**
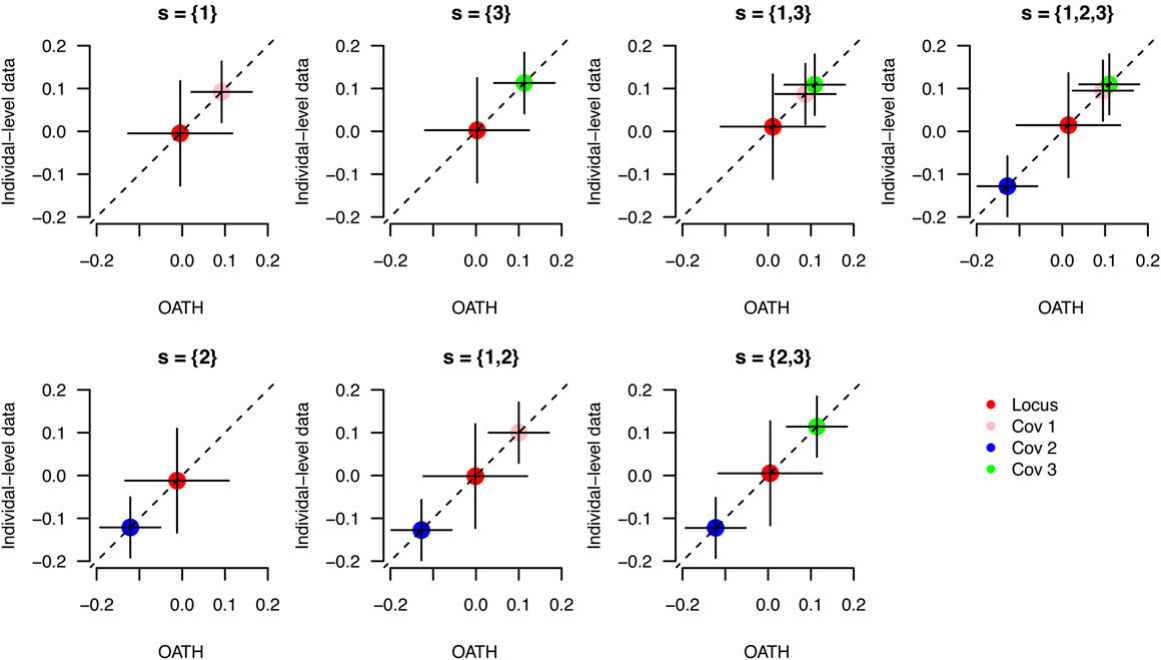
Demonstration of OATH for a single locus. Three covariates were simulated, and OATH generated seven models by including one, two, or three covariates. The subtitle for each plot indicates the set of covariates included. The *x*-axis represents the regression coefficients estimated from OATH, whereas the *y*-axis shows the regression coefficients for the corresponding model but estimated from the individual-level data. The vertical and horizontal lines across each point indicate the SE of the partial regression coefficient estimated via OATH and individual-level data, respectively. The subtitle in each panel indicates the covariates included. OATH, Open GWAS algoriTHm.

In order to demonstrate the OATH kernel, a single-locus analysis is shown. The MAF of the biallelic locus was 0.23, and the effect size was set to zero. Three covariates, each sampled from the standard normal distribution N(0,1), were simulated. The phenotype was sampled from N(0,1). The sample size was n=200. In this simulated sample, $\Phi =\left(\begin{array}{cc} \begin{array}{cc} 1 & -0.005\\ -0.005 & 0.338 \end{array} & \begin{array}{ccc} 0.092 & -0.121 & 0.113\\ -0.035 & 0.0063 & -0.0505 \end{array}\\ \begin{array}{cc} 0.092 & -0.035\\ -0.121 & 0.0063\\ 0.113 & -0.0505 \end{array} & \begin{array}{ccc} 1 & 0.0608 & 0.0506\\ 0.0608 & 1 & 0.0083\\ 0.0506 & 0.0083 & 1 \end{array} \end{array}\right),$ $\begin{array}{l} \Omega =\left[\begin{array}{cc} \begin{array}{cc} 0.338 & -0.035\\ -0.035 & 1 \end{array} & \begin{array}{cc} 0.0063 & -0.0505\\ 0.0608 & 0.0506 \end{array}\\ \begin{array}{cc} 0.0063 & 0.0608\\ -0.0505 & 0.0506 \end{array} & \begin{array}{cc} 1 & 0.0083\\ 0.0083 & 1 \end{array} \end{array}\right], \end{array}$ $\Lambda =\left[\begin{array}{cccc} 0.338 & & & \\ & 1 & & \\ & & 1 & \\ & & & 1 \end{array}\right],$and $\hat{b\prime }=\left[-0.0143,\;0.0922,\;-0.121,\;0.113\right].$ Including one, two, or three covariates, it generated seven possible models. The reproducibility of the partial regression coefficients estimated by OATH agreed well with those estimated from the individual-level data (Figure 2). An R script is available for this example at https://github.com/gc5k/OATH.

### Two Arabidopsis GWAS models

**Figure 3:**
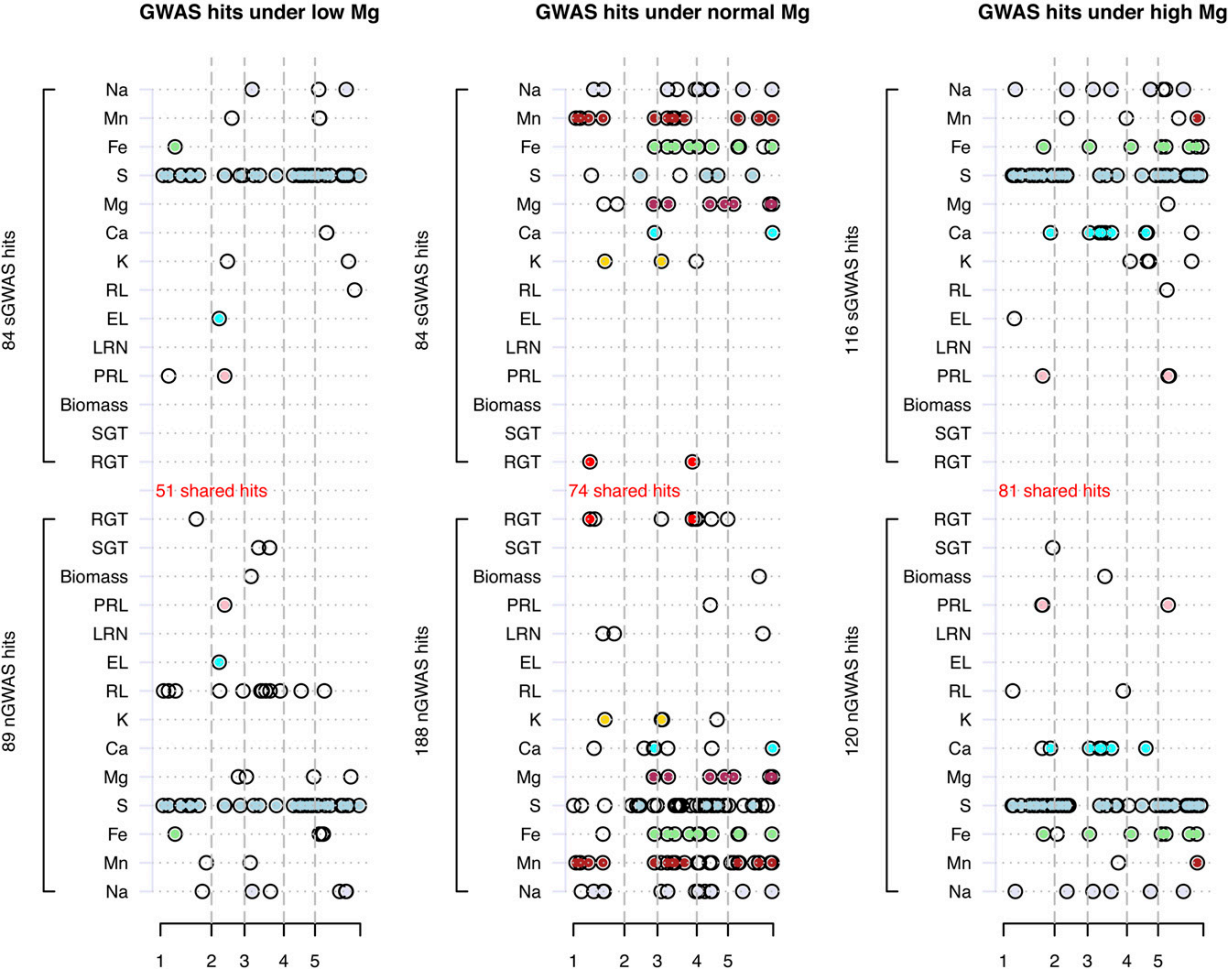
sGWAS and GWAS hits for 14 traits under three Mg conditions. The *x*-axis shows the chromosomal coordinates for *Arabidopsis*; the *y*-axis represents 14 traits. The GWAS hits observed in the saturated model (sGWAS), which was adjusted by the top five eigenvectors, are presented on the top; the hits observed in the naïve model (nGWAS) are represented in the bottom panel. GWAS hits are represented by black circles; and those hits shared by both nGWAS and sGWAS are filled with color. A total of 51, 74, and 81 GWAS hits were shared under low-, normal-, and high-Mg conditions, respectively. A GWAS hit was defined as $–\log_{10}\left(0.05/\#m\right)>6.5,$ in which the number of markers (#m) was 156,744. GWAS, genome-wide association studies; Mg, magnesium; nGWAS, naïve GWAS; sGWAS, saturated GWAS.

For these 295 *Arabidopsis* lines, we conducted a GWAS for each of the 42 phenotypes in the saturated GWAS models, which included the top five eigenvectors (Figure S7 in File S2). In contrast, we also conducted naïve/simple linear regressions (*i.e.*, no covariates) for these phenotypes, denoted as naïve GWAS (nGWAS) (Figure S8 in File S2). Under the 42 sGWAS, $\lambda_{\text{GC}},$ a metric measuring population stratification (Devlin and Roeder 1999), had a mean of 1.053±0.070, whereas that of the nGWAS was 1.151±0.128, indicating adjustment of the covariates in differentiating GWAS outcomes. For each phenotype, the correlations of the estimated β (additive genetic effect) and $–\log_{10}\left(p\right)$ between the sGWAS and the nGWAS were 0.828±0.099 and 0.905±0.053 , respectively (Figure S9 in File S2). Using $–\log_{10}\left(0.05/156744\right)=6.50$ as the nominal genome-wide significance threshold, the sGWAS had 284 hits in total; 84, 84, and 116 under the low-, normal-, and high-Mg conditions, respectively. The nGWAS had 397 hits in total; 89, 188, and 120 under the low-, normal-, and high-Mg conditions, respectively. Between the sGWAS and the nGWAS, 206 hits were shared (Figure 3). As demonstrated in this example, an alternative model could lead to different results, which might cause controversy over reproducibility.

### Reproducing sGWAS for Arabidopsis

In order to reproduce the sGWAS results for each SNP in the absence of shared original data, for each phenotype, the following NSS were used: the variance–covariance matrix of a phenotype, five eigenvectors, and information from 156,744 specific loci were also provided. Of note, the covariance matrix of the five eigenvectors was a diagonal matrix because the eigenvectors were mutually orthogonal.

**Figure 4:**
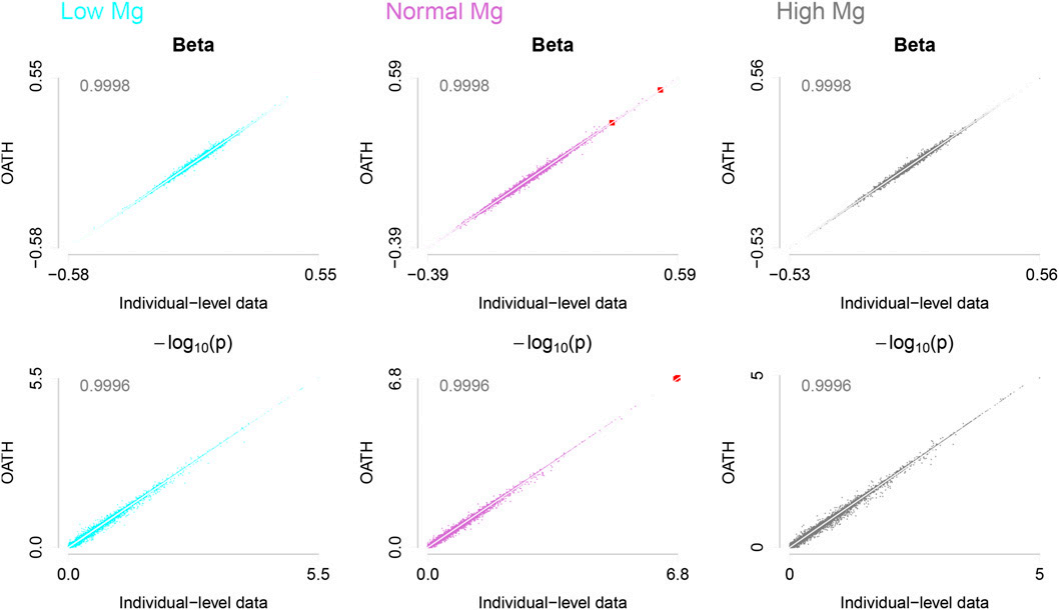
Reproducibility of sGWAS results using OATH with NSS for RTG under three Mg conditions. Each column represents β (top) and *p*-values (bottom) under low-, normal-, and high-Mg conditions. The *y*-axis represents the statistics from OATH synthesized from NSS, and the *x*-axis from the sGWAS; the red points are QTL detected in the sGWAS. Correlations are shown in the top left corner of each panel. GWAS, genome-wide association studies; Mg, magnesium; NSS, naïve summary statistics; OATH, Open GWAS algoriTHm; QTL, quantitative trait loci; RTG, days to root germination; sGWAS, saturated GWAS.

As expected, OATH synthesized the NSS as prepared above to reproduce the 42 sGWAS with high precision, as illustrated for days to root germination (RGT) (Figure 4), as well as for the 41 phenotypes (Figure S10 in File S2). For 14 traits under the normal-Mg condition, the consistency between the estimated β from OATH and those from the sGWAS was 1.00±0.00025 and 0.994±0.0014for–log(p). OATH found the same 284 hits that were found in the sGWAS. This indicated that, even without access to the individual-level data, OATH could retrospectively scrutinize the reported results. Furthermore, we also conducted individual-level data GWAS for these 295 *Arabidopsis* lines by including the top 10 eigenvectors, leading to $2^{10}$ possible outcomes for the association between a phenotype and a marker. OATH also almost perfectly reproduced the results (data not shown).

### Recovering underreported results for Arabidopsis

**Figure 5:**
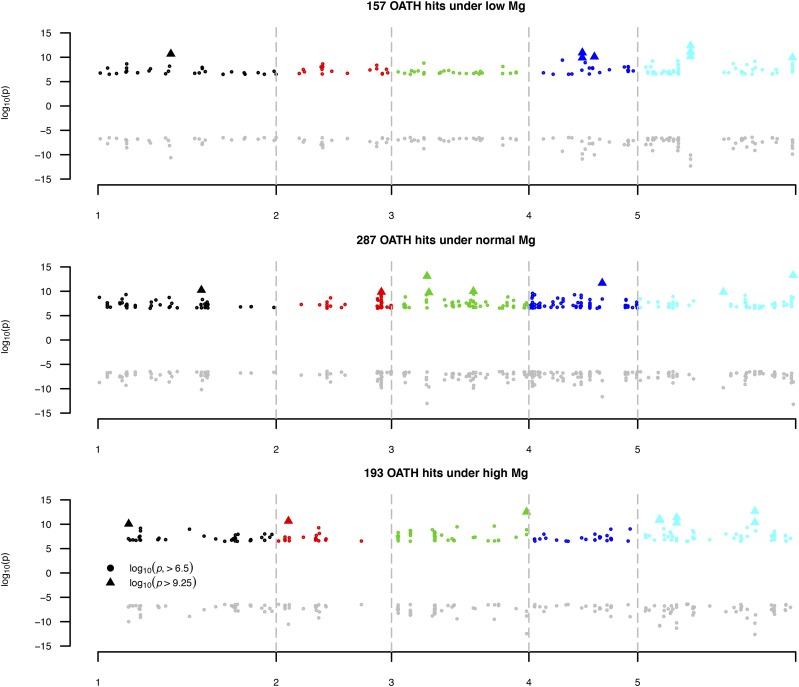
Validation of OATH hits by their exact models. A total of 637 OATH hits, which had a $\;–\log_{10}\left(p\right)>6.5,$ were found; 25 loci had a $–\log_{10}\left(p\right)>9.25.$ Within each panel, the positive part of the *y*-axis represents the $–\log_{10}\left(p\right)$ observed in OATH; the negative part of the *y*-axis is the corresponding $–\log_{10}\left(p\right)$ evaluated by their exact models using individual-level data from 295 *Arabidopsis* lines. OATH, Open GWAS algoriTHm.

These 295 *Arabidopsis* lines resulted in the generation of $2^{5}=32$ models, given all possible combinations of the five eigenvectors. With the inclusion or exclusion of certain eigenvectors, OATH was capable of synthesizing another 30 GWAS that had at least one of the five eigenvectors as covariates. For each of the 42 phenotypes, an OATH hit was claimed if a SNP had any of its 32 models in which the OATH $–\log_{10}\left(p\right)>6.50.$ OATH found 637 hits for 42 phenotypes; 163 hits were not found by either the sGWAS or the nGWAS. Of these 163 new hits, 25 had $–\log_{10}\left(p\right)>-\log_{10}\left(0.05/156744\;\times \;32\;\times \;42\right)=9.25\;,$ indicating a nominal overall significance under 42 phenotypes and 32 models. We validated these OATH hits by implementing their exact models using individual-level data from the 295 *Arabidopsis* lines; the consistency of the β and $–\log_{10}\left(p\right)$ was 0.9996±0.00016 and 0.990±0.0018 , respectively (Figure 5). Therefore, OATH found all possible underreported results with high consistency. These 637 OATH hits were found on 575 unique SNPs, for which 430 were within genes and 145 were between genic regions.

### In-depth evaluation of the GWAS hits for Arabidopsis

In the experimental design theory established by R. Fisher, a single high/low value, such as productivity in a field experiment, is often confounded by a combination of other factors (Fisher 1926) of little interest when compared with the values under different factors. Therefore, we further investigated whether the combination of the eigenvectors influenced each OATH hit.

**Figure 6:**
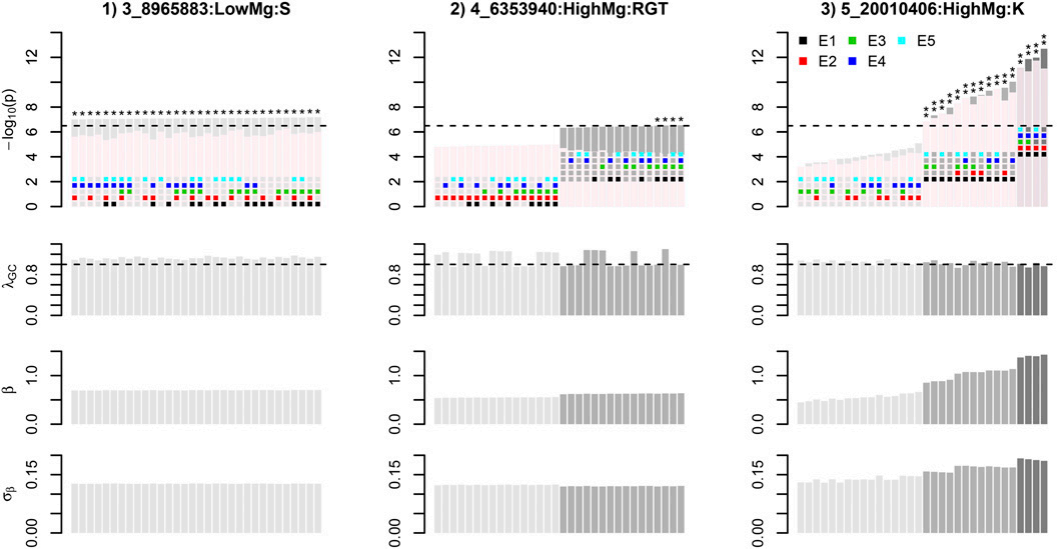
Evaluation of the modeling for three OATH hits. The top four rows represent $–{\text{log}}_{10}\left(p\right),$ $\lambda_{\text{GC}},$ β, and $\sigma_{\beta },$ estimated using 32 possible OATH models given five covariates for these three SNPs. $–\log_{10}\left(p\right),$ $\lambda_{\text{GC}},$ β, and $\sigma_{\beta }$ are in ascending order according to their $–\log_{10}\left(p\right).$ In the top row, the corresponding OATH models are denoted by colored squares, indicating the inclusion or exclusion of covariates; there are two bars, gray and pink, in each cluster, representing $–\log_{10}\left(p\right)$ with or without adjustment for $\lambda_{\text{GC}}.$ An asterisk indicates that this (SNP) is significant under the corresponding model without adjustment for $\lambda_{\text{GC}};$ three asterisks indicate that this SNP is significant under the corresponding model, both with and without adjustment for $\lambda_{\text{GC}}.$ OATH, Open GWAS algoriTHm; SNP, single nucleotide polymorphism.

For those 637 OATH hits, the smallest range of 32 $–\log_{10}\left(p\right),$ from 7.01 to 7.14, was found for SNP 3_8965883 (chromosome 3, 8,965,883 bp, and MAF = 0.0508) associated with sulfur under the low-Mg condition. SNP 3_8965883 was located within *RASPBERRY* 3 (*RSY3*), a gene related to embryogenesis (Apuya *et al.* 2002) (Table 2). Across the 32 models, its βs and SEs remained relatively stable (Figure 6).

**Table 2:** Three single nucleotide polymorphism (SNP) examples from *Arabidopsis* inbred lines

			Conservative Model				Powerful Model					Annotation				
SNP	A1	Freq.	Covariates*^a^*	$\beta_{1}$	$\sigma_{1}$	$-{\text{log}}_{10}\left(p\right)$		Covariates	$\beta_{1}$	$\sigma_{1}$	$-{\text{log}}_{10}\left(p\right)$	Treatment	Trait	Group #	*F*-Statistic	Gene
3_8965883	A	0.0508	−+−++	0.692	0.127	7.01		+−+−−	0.703	0.127	7.18	Low Mg	S	1		*RSY3*
4_6353940	T	0.0578	−+−−−	0.538	0.123	4.79		+−+++	0.636	0.121	6.54	High Mg	RGT	2	5168 (*p* < 1e−16)	*AT4G10200*
5_20010406	T	0.0508	−−+−+	0.449	0.13	3.19		++−+−	1.430	0.186	12.69	High Mg	K	3	434 (*p* < 1e−16)	*AT5G49350*

Each SNP had 32 models evaluated by Open GWAS algoriTHm (OATH) via naïve summary statistics. The smallest (conservative model) and the largest (powerful) $–\log_{10}\left(p\right)$ were tabulated. SNP, single nucleotide polymorphism; Freq., frequency; S, sulfur; RGT, days to root germination; K, potassium.

a “+” and “−” indicate inclusion and exclusion of the *j*th covariate.

In contrast, the largest range of $–\log_{10}\left(p\right),$ from 3.19 to 12.69 (Table 2), was found for SNP 5_200100406 (chromosome 5, 200,100,406 bp, and MAF = 0.058) associated with *K* under the high-Mg condition. SNP 5_200100406 was located within *AT5G49350*, a gene encoding glycine-rich protein (Tabata *et al.* 2000) (Table 2). Of its 32 $–\log_{10}\left(p\right),$ 16 were > 6.5. We partitioned its 32 sorted $–\log_{10}\left(p\right)$ into different groups if any two neighboring $–\log_{10}\left(p\right)$ differed by a unit. Its 32 $–\log_{10}\left(p\right)$ could be split into three groups (*F*-statistic = 434.06 and *p*-value < 1e−16). The four OATH models in the highest $–\log_{10}\left(p\right)$ group included the first, second, and fourth eigenvectors (Figure 6). Its βs were increased in the highest group but the corresponding SEs decreased, resulting in a much higher $–\log_{10}\left(p\right).$

In another example, SNP 4_6353940, associated with RGT under the high-Mg condition, had its 32 $–\log_{10}\left(p\right)$ partitioned into two groups via inclusion or exclusion of the second eigenvector (Figure 6). SNP 4_6353940 had a MAF of 0.0507 and was located within *AT4G10200*, a gene related to TTF-type zinc finger proteins with a HAT dimerization domain (Mayer *et al.* 1999) (Table 2). Inclusion or exclusion of the second eigenvector also resulted in two groups for the β. Among 637 OATH hits, this SNP had the most significant difference for its $–\log_{10}\left(p\right)$ group, and the *F*-statistic was 5168.142 (*p*-value < 1e−16).

An R script is available at https://github.com/gc5k/OATH for the demonstrated *Arabidopsis* analyses with OATH.

### Application of OATH to GWAMA

**Figure 7:**
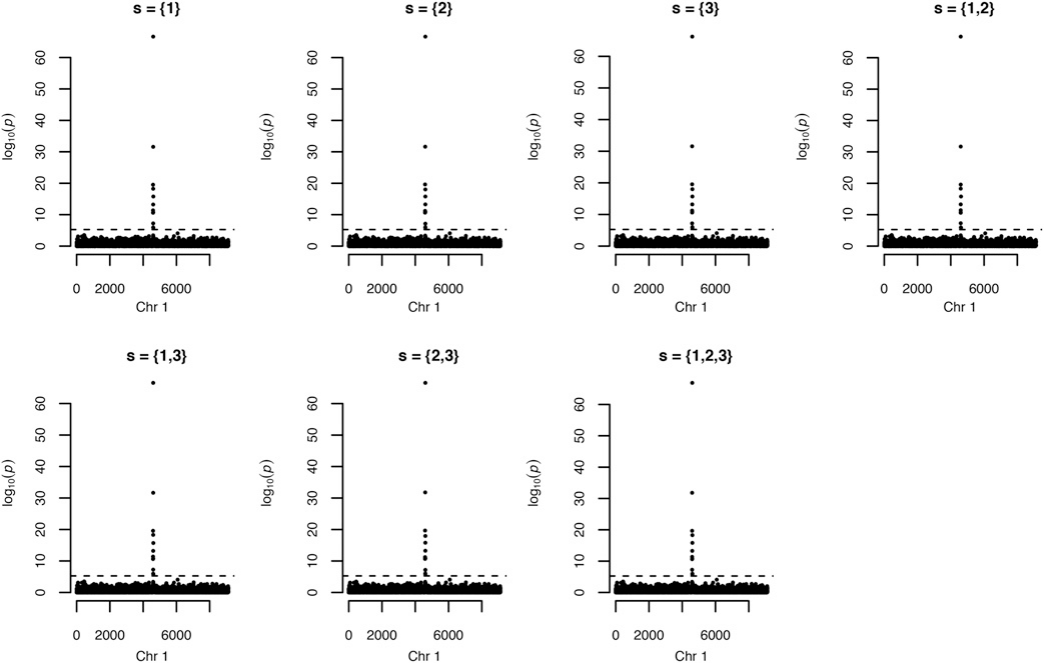
Genome-wide association meta-analyses (GWAMA) of the NA and SLE cohorts. The subtitle in each panel indicates a customized GWAMA. For example, s={1,3} indicates that the first and third eigenvectors are covariates for each of the two cohorts. The dashed line indicates the chromosome-wise threshold, given α=0.05.

Two Chinese GWAS datasets, the NA (Han *et al.* 2013) and SLE cohorts (Han *et al.* 2009), were used to confirm the utility of OATH for meta-analyses. From these two cohorts, 9124 common variants on chromosome 1 in both cohorts were analyzed in NA (3191 samples) and SLE (2309 samples), respectively. For both cohorts, the SNPs were aligned on the same reference alleles. SNP rs4144542 was set as the causal locus explaining 5% of the total phenotypic variation. Three eigenvectors were used as covariates. In order to mimic a real consortium-driven GWAMA, one author (HFZ) generated NSS for these two GWAS cohorts; another author (GBC), who was blind to the individual-level data, ran OATH and the meta-analyses. After receiving $\Phi_{i},$ the central hub synthesized seven corresponding $\beta_{i}^{*},$ given s={1},{2},{3},{1,2},{1,3},{2,3},{1,2,3}; consequently, meta-analyses could be implemented for each locus. As demonstrated in Figure 7, rs4144542 was successfully identified in all seven GWAMA analyses. Other loci had very similar estimated effects under these seven models.

An R script is available for this GWAMA demonstration at https://github.com/gc5k/OATH.

## Discussion

The scientific community is seeking reproducibility, and efforts have been made to improve reproducibility as well as transparency. Reproducibility may vary among studies; however, false discovery due to controversial or improper modeling can be monitored and even avoided, as demonstrated for the 295 *Arabidopsis* lines. Since the establishment of experimental design theory for field experiments (Fisher 1926), it has been known that a single outcome may be confounded, such as nutrition level factors. A high or low outcome makes little sense when it departs from its context, such as the conditions that led to the observed extreme values. In particular, as justification for the inclusion of covariates is controversial, variation in studies due to modeling makes reproducibility challenging (Aschard *et al.* 2015). As GWAS results are often reported using a particular model, the interpretation of a GWAS hit should be reasonably scrutinized, as demonstrated in this study.

We developed OATH and demonstrated its utility in GWAS of 295 *Arabidopsis* inbred lines. OATH successfully reproduced the GWAS results generated from a model with five covariates. In addition, underreported results, possibly generated by alternative models, were recovered. Given these comprehensive results, we could evaluate GWAS hits more thoroughly. As OATH is based on summary statistics, this implementation was compatible with GWAS data sharing policy, including those involving human subjects. For *Arabidopsis*, a typical admixed population, a linear mixed model technique provides an alternative solution (Korte *et al.* 2012); however, the complicated statistical properties of linear mixed models (Chen 2014, 2016; de los Campos *et al.* 2015) may be beyond OATH’s linear regression model capabilities.

Given the many possible ways to utilize OATH, GWAMA would most likely benefit from OATH integration. Using OATH, GWAMA would be more efficient at switching from one GWAS model to another whenever necessary, a procedure that often leads to logistical burden under a conventional GWAMA design. Many consortia that encourage open-source genetics have released GWAS summary statistics, such as GIANT and PGC. If those consortia would also release the naïve summary data required by OATH, efficiency and reproducibility can be dramatically boosted and the utility of the GWAS data maximized because the recovery of underreported GWAS discoveries becomes possible, as demonstrated in our study.

In summary, in line with the open-source movement, we believe that reproducibility, transparency, and in-depth evaluation of GWAS are possible or can be improved using the proposed method. OATH as a solution is simple and easily embedded into other applications, and the information technology seems mature enough for implementation. To facilitate application of the proposed method, we deposited OATH in Genetic Analysis Repository (GEAR; https://github.com/gc5k/GEAR). Three “one-click-for-all” R scripts for the demonstrated examples are available at https://github.com/gc5k/OATH.

## Supplementary Material

Supplemental material is available online at www.g3journal.org/lookup/suppl/doi:10.1534/g3.116.038877/-/DC1.
