# Supplementary material for: Reproduction and In-Depth Evaluation of Genome-Wide Association Studies and Genome-Wide Meta-analyses Using Summary Statistics
Source: G3 (Bethesda). 2017 Jan 24;7(3):943–52. doi: 10.1534/g3.116.038877 (PMC5345724; doi:10.1534/g3.116.038877)
Supplement: Supplementary file 1 [file 943FileS1.pdf]

Title: Reproducing and in-depth evaluation of genome-wide association studies and genome-wide meta-analyses using summary statistics

Authors: Yao-Fang Niu[*,1], Chengyin Ye[†], Ji He[§], Fang Han[‡], Long-Biao Guo[*], Hou-Feng Zheng[**,††], Guo-Bo Chen[§§,1]

Affiliations:

[*]State Key Laboratory of Rice Biology, China National Rice Research Institute, Hangzhou 310006, Zhejiang Province, China;

[†]Department of Health Management, College of Medicine, Hangzhou Normal University, Hangzhou 310021, Zhejiang, China;

[§]Department of Neurology, Peking University Third Hospital, Beijing 100191, China;

[‡]Department of Pulmonary, Critical Care Medicine, Peking University People's Hospital, Beijing 100044, China;

[**]Institute of Aging Research, School of Medicine, Hangzhou Normal University, Hangzhou 310021, Zhejiang, China;

[††]The Affiliated Hospital of Hangzhou Normal University, Hangzhou, Zhejiang, China, Hangzhou 310015, Zhejiang, China;

[§§]Evergreen Landscape and Architecture Studio, Hangzhou 310026, Zhejiang, China.

[1]YFN and GBC contributed equally.

Running title: Reproducible GWAS using summary statistics

Key words: GWAS, *Arabidopsis*, magnesium, transparency, reproducibility, naïve summary statistics, meta-analyses, GWAMA, GEAR

Correspondences:

HFZ: hfzheng@hznu.edu.cn

Postal address: Institute of Aging Research, School of Medicine, Hangzhou Normal University, Hangzhou 310021, Zhejiang, China.

GBC: chenguobo@gmail.com; (ORCID: 0000-0001-5475-8237)

Postal address: Evergreen Landscape and Architecture Studio, Xixi Road 562, Hangzhou 310007, Zhejiang Province, China.

Table of contents

Supplementary Note I: Background for Arabidopsis GWAS 3
- Magnesium 3
- Magnesium and other mineral nutrients 3
- Arabidopsis and GWAS 4
- Plant material 4
- Growth medium 5
- Growth conditions 5
- Phenotype analysis 6
- Analyses of mineral homeostasis 6

Supplementary Notes II: Open GWAS AlgoriTHm (OATH) 8
- Part I: the connection between a multiple regression and its corresponding simple regressions 8
- Part II: Open genome-wide Association algoriTHm (OATH) 9
  - GWAS model 9
  - Sufficient statistics for OATH 10
  - Customizing OATH for deep evaluation 10
  - Application for consortium-driven genome-wide association meta-analyses 11
- Part III: Linear regression and logistic regression for case-control GWAS. 12

Acknowledgements 13

Author contributions 14

References 15

Supplementary Note I: Background for Arabidopsis GWAS

Magnesium

Magnesium (Mg) is the 8th most abundant mineral element on earth and the fourth abundant mineral element in plants following nitrogen (N), potassium (K) and calcium (Ca) (Maguire and Cowan 2002). It is known to be an essential element for a large number of vital biochemical processes in all living organisms, including chlorophyll synthesis and many enzymatic reactions, including those involving ATPases, kinases and polymerases (Wilkinson, S.R., Welch, Ross M., Mayland, H.F., Grunes 1990; Cowan 2002; Hörtensteiner 2009), nucleotide metabolism (Igamberdiev and Kleczkowski 2001), photosynthetic carbon fixation (Lilley *et al.* 1974; Williams and Salt 2009), nucleic acid folding and the chemical catalysis of RNA splicing (Pyle 2002).

Magnesium in soils originates from source rock material containing various types of silicates and carbonates. However, long-term unbalanced crop fertilization practice neglecting Mg depletion of soils and cation competition and subsequent leaching lead to Mg deficiency in plants, decreased productivity and quality in agriculture practice worldwide (Bennett 1993). On the other hand, like other metals, Mg at high levels can deteriorate soil chemical and biological properties, and thus change the colonization and growth of plants. In particular, Mg-rich dust derived from mining and calcination has led to intense vegetation and soil damage (Kautz *et al.* 2001). Hazards of excessive Mg intake to human health include changes in mental status, nausea, diarrhea, appetite loss, muscle weakness, breathing difficulty, extremely low blood pressure, and irregular heartbeat (Swaminathan 2000). Concentrations of Mg ion in soil solutions lie between 0.125 and 8.5 mM, depending on soil texture and cation exchange capacity of the soil (Hariadi and Shabala 2004), the concentration of competing cations, the water availability or excessive leaching, crop cultivation and fertilizer regime (Broadley *et al.* 2008; Mikkelsen 2010). Abnormal Mg status in soil resulting from either Mg depletion or Mg excess is generally considered negative for the growth of the plants (Hermans, Vuylsteke, Coppens, Craciun, *et al.* 2010; Hermans, Vuylsteke, Coppens, Cristescu, *et al.* 2010; Visscher *et al.* 2010; Niu YF, Chai RS, Liu LJ, Jin GL, Liu M, Tang CX 2014). Thus both deficiency and excess of Mg should be taken into consideration during developing management strategies.

Magnesium and other mineral nutrients

Plants display an array of physiological responses to Mg availability, including morphological and architectural responses of the root system. It is documented that Mg^{2+} deficiency also impairs root growth

and thus the acquisition of mineral nutrients (Marschner *et al.* 1996). Mg availability affects the ionome by impacting on the uptake and distribution of other cations. Antagonistic and synergistic effects are largely reported between Mg, Ca and K in many plant species (Hermans *et al.* 2004; Ding *et al.* 2006; Karley and White 2009). In *Arabidopsis*, the most important increases in tissue concentration are observed mainly for the divalent cations, manganese (Mn), but also for Ca and iron (Fe) in leaves and for Ca and zinc (Zn) in roots after one week Mg deficiency treatment. However, in the past decade, the importance of Mg in plant development was underestimated, and therefore Mg was called 'a forgotten element' (Cakmak I. & Yazici A.M. 2010).

Arabidopsis and GWAS

Genome-wide association studies (GWAS) are a powerful tool for establishing correlation between phenotypes and genotypes. *Arabidopsis thaliana* has proved an almost ideal organism in which to conduct GWAS because it can be maintained as inbred lines via continued self-fertilization and more than 1000 inbred lines have been 'fully sequenced', removing the cost of genotyping for a set of lines that can be phenotyped over and over. Because more than 1300 distinct accessions have been genotyped for 250000 SNPs (Horton *et al.* 2012) all a researcher requires is the phenotype of several hundred lines for a trait of interest. In addition to the land mark proof-of-concept GWAS study of 107 phenotypes (Atwell *et al.* 2010) numerous other traits including glucosinolate level (Chan *et al.* 2011) shade avoidance (Filiault and Maloof 2012), flowering time (Li *et al.* 2010), primary root length (Mouchel *et al.* 2004; Loudet *et al.* 2005; Sergeeva *et al.* 2006), total root size (Fitz Gerald 2005) and root systems architecture(Rosas *et al.* 2015), heavy metal (Chao *et al.* 2012), salt tolerance (Baxter *et al.* 2010), iron deficient (Stein and Waters 2012), potassium starvation (Kellermeier *et al.* 2013), local adaptation (Fournier-Level *et al.* 2011), low water potential-induced proline accumulation (Verslues *et al.* 2014), flavonol and anthocyanin metabolism (Schulz *et al.* 2015) and telomere length (Fulcher *et al.* 2015) have been successfully analyzed. Though natural variation within *Arabidopsis* has been the basis for above studies on plant morphology, physiology, and development as well as stress response, the responses to Mg supply have not been dissected.

Plant material

Seeds of all 295 lines were derived from the *Arabidopsis* Biological Resources Center stock center with accession CS76636, CS76427, CS78885, CS22660. Wt, Ws-1, Ws-2 and En-2 from the Nottingham *Arabidopsis* Stock Centre (http://nasc.nott.ac.uk) were also included in the analysis. Distribution of over 295 *Arabidopsis* accessions collected from the wild and available in the stock center or soon-to be-released

collections. Most of these 295 lines were originated from European nations, such as Sweden, Germany, France, Czech Republic, United Kingdom, and from North America and Middle Asia, representing the sampling strategy of the 1,307 worldwide accessions.

Growth medium

The Mg basal medium (Normal Mg), which was used as control, contained (µM) 1500 KNO_3, 500 NaH_2PO_4, 1000 $CaCl_2$, 250 $(NH_4)_2SO_4$, 1000 $MgSO_4$, 1250 Na_2SO_4, 25 Fe-EDTA, 10 H_3BO_3, 0.5 $MnSO_4$, 0.5 $ZnSO_4$, 0.1 $CuSO_4$ and 0.1 $(NH_4)_6Mo_7O_{24}$ (Hoagland and Arnon 1950). Mg treatments were achieved by altering the concentrations of $MgSO_4$ in the basal medium. Thus the low Mg medium contained 1 µM $MgSO_4$ while high Mg medium 10,000 µM $MgSO_4$. Meanwhile, the medium with lower concentrations of Mg were supplied by sodium sulfate so that decreasing the difference of SO_4^{2-} concentration among the treatments. Though small differences were present among the Mg treatments, such differences in SO_4^{2-} ion did not affect morphogenesis of *Arabidopsis* (Gruber *et al.* 2013; Niu YF, Chai RS, Liu LJ, Jin GL, Liu M, Tang CX 2014).

The pH of the growing media was adjusted to pH 5.8 with MES (N-morpholino) ethane-sulphonic acid)-KOH buffer before autoclaving. The concentrations of Mg in the control were 1000 µM, that has been adopted for *Arabidopsis* growth by many plant biologists (Lanquar *et al.* 2009; Costa *et al.* 2013; Yang *et al.* 2013). In addition, our preliminary experiment showed that 10, 000 µM $MgSO_4$ did not cause any toxicity symptoms during the experimental period (Niu YF, Chai RS, Liu LJ, Jin GL, Liu M, Tang CX 2014; Niu *et al.* 2015).

Growth conditions

To reduce maternal effects prior to phenotyping, natural accessions were grown for one generation during 2015 under controlled greenhouse conditions at the ZiJinGang campus in Zhejiang university (N30º18´25, E120º04´54). For surface sterilization, *Arabidopsis thaliana* seeds of various accessions were placed for 1 h in opened 200-µL PCR tubes in a sealed box containing chlorinegas generated from 13mL of 10% sodium hypochlorite and 350 µL of 37% hydrochloric acid. Sterile seeds were then put on the surface of 30 mL agar media, containing 1.2% (w/v) agar and 0.6 % (w/v) sucrose (A-1296; Sigma-Aldrich; http://www.sigmaaldrich.com) in 10×10-cm^2 plates with grid schematic engraved below the plate. Plates were positioned in racks and oriented in a vertical position, and were kept at 4 °C for 48 h in the dark for seed stratification. Thereafter, the racks containing the plates were transferred to a growth chamber under a 10 h light/14 h dark photoperiod at constant temperature of 22 °C, 60 % relative humidity and light intensity

of 120 μmol photons m^{-2} s^{-1}. Twelve seedlings were grown in each plate and each treatment received at least four independent replicates. The racks were removed to the image acquisition room once per day and then immediately returned to the growth chamber. Throughout the experiments, the plate position within the box and box position in the growth chamber were rerandomized every day. For assays on agar plates, studies were performed on 8-d-old plants; that is, at an early stage of their stability and homogeneity growth phase. Moreover, many excellent publications adopted 6- to 8-d-old *Arabidopsis* seedlings under medium for determining *Arabidopsis* morphology, physiology, and development as well as stress response (Weber *et al.* 2007; Czarnecki *et al.* 2011; Greco *et al.* 2012).

Phenotype analysis

For phenotypic analysis of Arabidopsis accessions, four or five seeds were sown in equal distance on 10×10-cm^2 square petri dishes containing low Mg, normal Mg or high Mg medium, respectively. Seeds that had not germinated at 6 day were discarded from further analysis, resulting in approximately four seedlings analyzed per genotype per condition. Seedlings were photographed with a high-resolution digital camera (Sony RX100, Japan) per day for determination root and shoot germination. Root or shoot germination, the number of days from seeding until emergence with more than half seedling have first radicle or cotyledon, respectively. Meanwhile, photographs after 8 d of treatment were analyzed and quantified for phenotype using the public domain image analysis program Image J version 1.43 (http://rsb.info.nih.gov/ij/) (Niu *et al.* 2015). Length of primary root, rosette diameter and epicotyl were determined across the median seedling using Image J. Lateral root number was determined by counting the number of true roots (>1 mm long lateral root primordia) per primary root. The scale was set for the picture within the program. For each condition, a represent biological sample was measured on independent sample accession from four different plants at the same growth stage and the time of sampling was the end of the light period of day 8.

Root germination, shoot germination and lateral root number data were showed as the value obtained in low Mg or high Mg treatment minus those under normal Mg treatment. The values of primary root, epicotyl length and rosette width length for low Mg or high Mg treatment were then divided by values obtained with normal Mg treatment.

Analyses of mineral homeostasis

After 8-d growth at various Mg concentrations, plants were harvested; all fully-expanded and non-lesioned seedlings were collected from each accession, and weighed to obtain the fresh weight measurements. The

results are the average value across all available replicates. Seedlings were washed thoroughly with ultrapure water and dried in an oven at 75 °C for 12 h. Then the dried root and shoot samples were wet-digested in the concentrated HNO_3/H_2O_2 at 90, 120 and 140 °C for 2 h, respectively, until there was no brown fume, and then further digested at 180 °C until the digest became clear. Concentrations of potassium (K), calcium (Ca), magnesium (Mg), sulfur (S), iron (Fe), manganese (Mn) and sodium (Na) in the digests were analyzed by ICP-MS (Inductively coupled plasma mass spectrometer, Agilent 7500a, USA), and were calculated on a basis of fresh-weight (FW) of seedlings. The results are the average value across all available replicates. Biomass and nutrient concentration data under low Mg or high Mg were calculated as the ratio of the treatment value (low Mg or high Mg) divided by the normal in which seeds were germinated in normal Mg.

Supplementary Notes II: Open GWAS AlgoriTHm (OATH)

Part I: the connection between a multiple regression and its corresponding simple regressions

For a multiple regression model

$\boldsymbol{y} = \beta_1\boldsymbol{x}_1 + \beta_2\boldsymbol{x}_2 + \cdots + \beta_m\boldsymbol{x}_m + e$ [A.1]

$\boldsymbol{\beta}' = (\hat{\beta}_1, \hat{\beta}_2, \ldots, \hat{\beta}_m)$ is least-squares estimate for the partial regression coefficients of the multiple regression model A.1. For each x_i, it has

$$cov(y, x_2) = \hat{\beta}_1\sigma_{x_1,x_2} + \hat{\beta}_2\sigma_{x_1}^2 + \cdots + \hat{\beta}_m\sigma_{x_m,x_2}$$

$$\vdots$$

$$cov(y, x_m) = \hat{\beta}_1\sigma_{x_1,\square m} + \hat{\beta}_2\sigma_{x_2,x_m} + \cdots + \hat{\beta}_m\sigma_{x_m}^2$$

and its general pattern suggests

$$\begin{pmatrix} \sigma_{x_1}^2 & \sigma_{x_1,x_2} & \cdots & \sigma_{x_1,x_m} \\ \sigma_{x_2,x_1} & \sigma_{x_2}^2 & \cdots & \sigma_{x_2,x_m} \\ \vdots & \vdots & \ddots & \vdots \\ \sigma_{x_m,x_1} & \sigma_{x_m,x_2} & \cdots & \sigma_{x_m}^2 \end{pmatrix} \begin{pmatrix} \hat{\beta}_1 \\ \hat{\beta}_2 \\ \vdots \\ \hat{\beta}_m \end{pmatrix} = \begin{pmatrix} \sigma_{y,x_1} \\ \sigma_{y,x_2} \\ \vdots \\ \sigma_{y,x_m} \end{pmatrix} \quad \text{[A.2]}$$

Of note, the right side of A.2 can be written as $\begin{pmatrix} \sigma_{y,x_1} \\ \sigma_{y,x_2} \\ \vdots \\ \sigma_{y,x_m} \end{pmatrix} = \begin{pmatrix} \sigma_{x_1}^2 & & & \\ & \sigma_{x_2}^2 & & \\ & & \ddots & \\ & & & \sigma_{x_m}^2 \end{pmatrix} \begin{pmatrix} \hat{b}_1 \\ \hat{b}_2 \\ \vdots \\ \hat{b}_m \end{pmatrix}$, because

$\hat{b}_j = \sigma_{y,x_j/\sigma_{x_j}^2}$ – the least-squares estimate for $\boldsymbol{y} = b_j\boldsymbol{x}_j + e$. So A.2 can be rewritten as

$$\begin{pmatrix} \sigma_{x_0}^2 & \sigma_{x_0,x_1} & \cdots & \sigma_{x_0,x_1} \\ \sigma_{x_1,x_0} & \sigma_{x_1}^2 & \cdots & \sigma_{x_1,x_m} \\ \vdots & \vdots & \ddots & \vdots \\ \sigma_{x_m,x_0} & \sigma_{x_m,x_1} & \cdots & \sigma_{x_m}^2 \end{pmatrix} \begin{pmatrix} \hat{\beta}_0 \\ \hat{\beta}_1 \\ \vdots \\ \hat{\beta}_m \end{pmatrix} = \begin{pmatrix} \sigma_{x_0}^2 & & & \\ & \sigma_{x_1}^2 & & \\ & & \ddots & \\ & & & \sigma_{x_m}^2 \end{pmatrix} \begin{pmatrix} \hat{b}_0 \\ \hat{b}_1 \\ \vdots \\ \hat{b}_M \end{pmatrix}$$

If these matrices and vectors from left to right are abbreviated as $\boldsymbol{\Omega}$, $\boldsymbol{\beta}$, $\boldsymbol{\Lambda}$, and $\mathbf{b}$, respectively, and after pre-multiplying $\boldsymbol{\Omega}^{-1}$ at both sides it turns out

$\hat{\boldsymbol{\beta}} = \boldsymbol{\Omega}^{-1}\boldsymbol{\Lambda}\hat{\mathbf{b}}$ [A.3]

as showed in the main text.

Alternative routes are possible, such as from least squares or from mixed model equations, to establish the connection between a multiple regression and its corresponding simple linear regressions (Robinson 1991; Yang *et al.* 2012).

Part II: Open genome-wide Association algoriTHm (OATH)

GWAS model

A multiple regression model for a saturated GWAS analysis is written as (for the ease of discussion, all variables are centered)

$$\boldsymbol{y} = \beta_i^* \boldsymbol{x}_i + \beta_1 \boldsymbol{z}_1 + \cdots + \beta_m \boldsymbol{z}_m + e \qquad [1]$$

in which $\boldsymbol{y}$ is for the centered observed phenotype of n individuals, $\boldsymbol{x}_i$ codes for the counts of the reference alleles at the ith locus, $\boldsymbol{z}_j$ is the jth covariate, and e is the residual. β_i^* is the effect size for the marker, β_j is the partial regression coefficient. Denote $\boldsymbol{X}_i = [x_i^* : z_1 : \ldots : z_m]$, and $\boldsymbol{\beta}_i' = [\beta_i^*, \beta_1, \beta_2, \ldots, \beta_m]$. For GWAS, only β_i^* is of interest, and the partial regression coefficients for covariates are often treated as nuisance parameters.

The least-squares estimator for the partial regression coefficients is $\boldsymbol{\beta}_i = \boldsymbol{\Omega}_i^{-1} \boldsymbol{X}_i \boldsymbol{y}$, in which $\boldsymbol{\Omega}_i = \boldsymbol{X}_i' \boldsymbol{X}_i$. Both $\boldsymbol{X}_i$ and $\boldsymbol{y}$ are individual-level data in the estimator. With inclusion of exclusion of certain covariates in $\boldsymbol{X}_i$, there are $c = \sum_{t=0}^{m} \binom{m}{t}$ possible ways to tailor $\boldsymbol{X}_i$, and consequently c possible estimators for $\boldsymbol{\beta}_i$ and $\hat{\beta}_i^*$. Given limit data access for individual-level data, it is hardly to recover the alternative estimates for alternative $\hat{\beta}_i^*$s if they are underreported in the original study.

Nevertheless, it exists an alternative estimator for $\boldsymbol{\beta}$ (see **Part I**), which is abbreviated as OATH (**O**pen **G**WAS algori**TH**m)

$$\widehat{\boldsymbol{\beta}}_i = \boldsymbol{\Omega}_i^{-1} \boldsymbol{\Lambda}_i \hat{\mathbf{b}}_i \qquad [2]$$

in which $\boldsymbol{\Lambda}_i$ is the diagonal of $\boldsymbol{\Omega}_i$. $\hat{\mathbf{b}}_i' = [\hat{b}_i^*, \hat{b}_1, \hat{b}_2, \ldots, \hat{b}_m]$, and each element is the regression coefficient from the array of simple regressions

$$\begin{cases} \boldsymbol{y} = b_i^* \boldsymbol{x}_i + e_i \\ \boldsymbol{y} = b_1 \boldsymbol{z}_1 + e_1 \\ \boldsymbol{y} = b_2 \boldsymbol{z}_2 + e_2 \\ \vdots \\ \boldsymbol{y} = b_m \boldsymbol{z}_m + e_m \end{cases}$$

. Eq 2 indicates that the joint least-squares estimate $\widehat{\boldsymbol{\beta}}_i$ can be synthesized with summary statistics. The sampling variance-covariance matrix of $\boldsymbol{\beta}_i$ is

$$\hat{\sigma}_{\beta_i}^2 = \left(\frac{\sigma_y^2 - \widehat{\boldsymbol{\beta}}_i' \boldsymbol{\Lambda}_i \hat{\mathbf{b}}_i}{n-m} \right) \boldsymbol{\Omega}_i^{-1} \qquad [3]$$

In addition, OATH can be applied to case-control studies, but an approximation (see text blew).

Sufficient statistics for OATH

The sufficient statistics for Eq 2 & 3 are capsulated in $\mathbf{\Phi}_i = \begin{pmatrix} \sigma_y^2 & \sigma_{y,x_i} & \sigma_{y,z_1} & \cdots & \sigma_{y,z_m} \\ \sigma_{x_i,y} & \sigma_{x_i}^2 & \sigma_{x_i,x_1} & \cdots & \sigma_{x_i,z_m} \\ \sigma_{z_1,y} & \sigma_{z_1,x_i} & \sigma_{z_1}^2 & \cdots & \sigma_{z_1,z_m} \\ \vdots & \vdots & \vdots & \ddots & \vdots \\ \sigma_{z_m,y} & \sigma_{z_m,x_i} & \sigma_{z_m,x_1} & \cdots & \sigma_{z_m}^2 \end{pmatrix}$ the variance-covariance matrix of all variables in Eq 1. $\mathbf{\Phi}_i$ can be decomposed into two matrices,

$$\mathbf{\Phi}_i = \mathbf{G} + \mathbf{L}_i = \begin{pmatrix} \sigma_y^2 & & \sigma_{y,z_1} & \cdots & \sigma_{y,z_m} \\ & & & \cdots & \\ \sigma_{z_1,y} & & \sigma_{z_1}^2 & \cdots & \sigma_{z_1,z_m} \\ \vdots & \vdots & \vdots & \ddots & \vdots \\ \sigma_{z_m,y} & & \sigma_{z_m,x_1} & \cdots & \sigma_{z_m}^2 \end{pmatrix} + \begin{pmatrix} & \sigma_{y,x_i} & & \cdots & \\ \sigma_{x_i,y} & \sigma_{x_i}^2 & \sigma_{x_i,z_1} & \cdots & \sigma_{x_i,z_m} \\ & \sigma_{z_1,x_i} & & & \\ & \vdots & & & \\ & \sigma_{z_m,x_i} & & \cdots & \end{pmatrix}$$

in which $\mathbf{G}$ is generic to each locus, and $\mathbf{L}_i$ is locus-specific for x_i^*. As $\mathbf{L}_i$ is a sparse symmetric matrix, its second row/column, denoting $\boldsymbol{l}_i$, is sufficient to represent all information. By dropping off the first row and the first column of $\mathbf{\Phi}_i$, it gets $\mathbf{\Omega}_i = \begin{pmatrix} \sigma_{x_i}^2 & \sigma_{x_i,z_1} & \cdots & \sigma_{x_i,z_m} \\ \sigma_{z_1,x_i} & \sigma_{z_1}^2 & \cdots & \sigma_{z_1,z_m} \\ \vdots & \vdots & \ddots & \sigma_{z_1,z_m} \\ \sigma_{z_m,x_i} & \sigma_{z_m,z_1} & \cdots & \sigma_{z_m}^2 \end{pmatrix}$. $\hat{\mathbf{b}}_i = [\frac{\sigma_{y,x_i}}{\sigma_{x_i}^2}, \frac{\sigma_{y,z_1}}{\sigma_{z_1}^2}, \frac{\sigma_{y,z_2}}{\sigma_{z_2}^2}, \ldots, \frac{\sigma_{y,z_m}}{\sigma_{z_m}^2}]$ can be estimated via the elements in $\mathbf{\Phi}_i$ too (**Figure 1**). So all elements necessary for Eq 2 & 3 can be found in $\mathbf{\Phi}_i$. As all elements in $\mathbf{\Phi}_i$ are merely variance and covariance, we also call them naïve summary statistics (NSS) in the text below.

Customizing OATH for deep evaluation

Furthermore, by including certain covariates for $\mathbf{\Phi}_{i.s}$, in which s indicates the set of covariates included for Eq 1, Eq 2 can be customized into any of the c models,

$$\widehat{\boldsymbol{\beta}}_{i.s} = \mathbf{\Omega}_{i.s}^{-1}\mathbf{\Lambda}_{i.s}\hat{\mathbf{b}}_{i.s} \qquad [4]$$

Given m covariates, there are c possible forms for s, for example $s = \{1, m\}$, corresponding to $\boldsymbol{y} = \beta_i^*\boldsymbol{x}_i + \beta_1\boldsymbol{z}_1 + \beta_m\boldsymbol{z}_m + e$ and $\mathbf{\Phi}_{i.s} = \begin{pmatrix} \sigma_y^2 & \sigma_{y,x_i} & \sigma_{y,z_1} & \sigma_{y,z_m} \\ \sigma_{x_i,y} & \sigma_{x_i}^2 & \sigma_{x_i,y} & \sigma_{x_z,y} \\ \sigma_{z_1,y} & \sigma_{z_1,x_i} & \sigma_{x_1}^2 & \sigma_{z_1,z_m} \\ \sigma_{z_m,y} & \sigma_{z_m,x_i} & \sigma_{z_m,z_1} & \sigma_{z_m}^2 \end{pmatrix}$, then Eq 4 becomes

$$\begin{pmatrix} \hat{\beta}_i^* \\ \hat{\beta}_1 \\ \hat{\beta}_m \end{pmatrix} = \begin{pmatrix} \sigma_{x_i}^2 & \sigma_{x_i,z_1} & \sigma_{x_i,z_m} \\ \sigma_{z_1,x_i} & \sigma_{z_1}^2 & \sigma_{x_1,z_m} \\ \sigma_{z_m,x_i} & \sigma_{z_m,x_1} & \sigma_{z_m}^2 \end{pmatrix}^{-1} \begin{pmatrix} \sigma_{x_i}^2 & & \\ & \sigma_{z_1}^2 & \\ & & \sigma_{z_m}^2 \end{pmatrix} \begin{pmatrix} \hat{b}_i^* \\ \hat{b}_1 \\ \hat{b}_m \end{pmatrix}.$$

Sampling variance for $\widehat{\boldsymbol{\beta}}_s$ can be estimated correspondingly.

$$\hat{\sigma}^2_{\boldsymbol{\beta}_{i.s}} = \left(\frac{\sigma_y^2 - \hat{\boldsymbol{\beta}}'_{i.s}\boldsymbol{\Lambda}_{i.s}\hat{\mathbf{b}}_{i.s}}{n - \mathcal{s}}\right)\boldsymbol{\Omega}^{-1}_{i.s} \qquad [5]$$

in which $\mathcal{s}$ is the number of elements in s.

So recovering underreported results for GWAS is possible if the summary statistics $\boldsymbol{\Phi}_i$ is simply provided. As the redundancy information in $\boldsymbol{\Phi}_i$ can be further squeezed out, for a GWAS of M loci, the generic matrix $\mathbf{G}$ and M vectors for $\boldsymbol{l}_i$ of each locus provide all information, sufficient statistics, to OATH, which promises deep evaluation of GWAS results.

Application for consortium-driven genome-wide association meta-analyses

In GWAMA, the effect size is often synthesized via inverse-variance estimator, or written in the generalized linear regression below

$$\boldsymbol{B}_i^* = \mu_i + e \qquad [6]$$

in which $\boldsymbol{B}_i^{*\prime} = [\beta^*_{i(1)}, \beta^*_{i(2)}, \beta^*_{i(3)}, \dots, \beta^*_{i(K)}]$. $\beta^*_{i(j)}$ is the additive effect estimated for the ith locus estimated from the jth cohort. μ_i is the mean of the regression, the estimated effect of the locus. $e \sim N(0, \omega)$, and $\omega = \begin{pmatrix} \frac{1}{\sigma^2_{\beta^*_{i(1)}}} & & \\ & \ddots & \\ & & \frac{1}{\sigma^2_{\beta^*_{i(K)}}} \end{pmatrix}$ is the weighted residual. In conventional consortium-driven GWAMA, each cohort run a GWAS model, such as Eq 1, and sends $\hat{\beta}_i^*$ and $\hat{\sigma}^2_{\beta_i^*}$ to the GWAMA central hub. However, if each cohort sends $\boldsymbol{\Phi}_i$ to the central hub, the whole GWAMA will gain more flexible. For example, when the GWAMA consortium wants to drop off the 5th covariate, $\boldsymbol{B}^*_{i.s} = [\beta^*_{i(1).s}, \beta^*_{i(2).s}, \beta^*_{i(3).s}, \dots, \beta^*_{i(K).s}]$, in which $\beta^*_{i(1).s}$, $s = \{1,2,3,4\}$, can be synthesized using Eq 5. So, in general, Eq 6 can be generalized as

$$\boldsymbol{B}^*_{i.s} = \mu_i + e \qquad [7]$$

Rather than letting each cohort run GWAS and upload the summary statistics such as $\hat{\beta}^*_{i(k)}$ and $\hat{\sigma}^2_{i(k)}$ again, a logistic expensive procedure, a consortium-driven GWAMA can increase the efficiency by running OATH at the analysis central hub using NSS $\boldsymbol{\Phi}$ provided by each cohort.

Part III: Linear regression and logistic regression for case-control GWAS.

Given the prevalence of K for a case-control study, and a biallelic locus, we can conduct a linear regression $y = a + bx + e$.

The expectation of the regression coefficient is $b = \frac{cov(x,y)}{var(x)}$. Under the Hardy-Weinberg equilibrium,

	x		
y	AA (2)	Aa (1)	aa (0)
1	$p_1^2 K$	$2p_1q_1K$	$q_1^2 K$
0	$p_2^2(1-K)$	$2p_2q_2(1-K)$	$q_2^2(1-K)$

$E(y) = K,\ E(x) = 2Kp_1 + 2(1-K)p_2; E(xy) = 2Kp_1^2 + 2p_1q_1K = 2Kp_1.$

$$cov(x,y) = E(xy) - E(x)E(y) = 2K(1-K)(p_1 - p_2)$$

$$var(x) = K2p_1q_1 + (1-K)2p_2q_2 + K[p_1 - E(X)]^2 + (1-K)[p_2 - E(X)]^2$$
$$= 2p_1q_1K + 2p_2q_2(1-K) + 4K(1-K)(p_1 - p_2)^2$$

When there is no difference between p_1 and p_2, $var(x) = 2p_1q_1$.

$b = \frac{cov(x,y)}{var(x)} = \frac{2K(1-K)(p_1-p_2)}{2p_1q_1K+2p_2q_2(1-K)+4K(1-K)(p_1-p_2)^2} \approx \frac{2K(1-K)(p_1-p_2)}{2p_1q_1K+2p_2q_2(1-K)} \approx \frac{K(1-K)(p_1-p_2)}{p_1q_1}$, if p_1 is close to p_2.

Also, in a logistic regression, $\text{logit}\left(\frac{\text{K}}{1-\text{K}}\right) = \alpha + \beta x + e$, in which $\beta = \log(OR)$.

The odds ratio is $OR = \frac{p_1(1-p_2)}{p_2(1-p_1)}$, and when OR is not too far away from 1, $\beta = \log(OR) \approx 1 - OR = \frac{p_1 - p_2}{p_2(1-p_1)}$.

So, $\frac{b}{\beta} \approx K(1-K)$, an approximate linear relationship exists between these two estimates.

Acknowledgements

This work was supported by the Natural Science Foundation of China (31601277 to YFN; 81402762 to CY; 81601105 to JH; 81501145 to HFZ), the ninth special grants for post-doctoral (2016T90156 to YFN) and the China Postdoctoral Science Foundation (2014M551754 and 2015M581216 to YFN), the Zhejiang Provincial Natural Science Foundation for Distinguished Young Scholars of China (LR17H070001 to HFZ). We thank Hangzhou Guhe Information and Technology Co., Ltd for the help with bioinformatics assistance.

Author contributions

GBC and YFN conceived and designed the study. GBC developed the theory, performed the *Arabidopsis* GWAS analysis, GWAMA, and developed GEAR::OATH. YFN performed the material collection and *Arabidopsis* experimental operations, wrote the protocol for the material growth, and conducted phenotype analysis. FH and HFZ cleaned and provided the naïve summary statistics of NAcohort and SLEcohort. CY prepared the R scripts for online demonstration. GBC and YFN wrote the manuscript. JH and LBG contributed to the improving of the study and manuscript.

References

Atwell, S., Y. S. Huang, B. J. Vilhjálmsson, G. Willems, M. Horton *et al.*, 2010 Genome-wide association study of 107 phenotypes in Arabidopsis thaliana inbred lines. Nature 465: 627–31.

Baxter, I., J. N. Brazelton, D. Yu, Y. S. Huang, B. Lahner *et al.*, 2010 A Coastal Cline in Sodium Accumulation in Arabidopsis thaliana Is Driven by Natural Variation of the Sodium Transporter AtHKT1;1. PLoS Genet. 6: e1001193.

Bennett, W., 1993 Nutrients deficiencies & toxicities in crop plants. Am. Phythopathological Soc. Press.

Broadley, M. R., J. P. Hammond, G. J. King, D. Astley, H. C. Bowen *et al.*, 2008 Shoot calcium and magnesium concentrations differ between subtaxa, are highly heritable, and associate with potentially pleiotropic loci in Brassica oleracea. Plant Physiol. 146: 1707–1720.

Cakmak I. & Yazici A.M., 2010 Magnesium: a forgotten element in crop production. Better Crop. 94: 23–25.

Chan, E. K. F., H. C. Rowe, J. A. Corwin, B. Joseph, and D. J. Kliebenstein, 2011 Combining Genome-Wide Association Mapping and Transcriptional Networks to Identify Novel Genes Controlling Glucosinolates in Arabidopsis thaliana. PLoS Biol. 9: e1001125.

Chao, D.-Y., A. Silva, I. Baxter, Y. S. Huang, M. Nordborg *et al.*, 2012 Genome-wide association studies identify heavy metal ATPase3 as the primary determinant of natural variation in leaf cadmium in Arabidopsis thaliana. PLoS Genet. 8: e1002923.

Costa, M., M. S. Nobre, J. D. Becker, S. Masiero, M. I. Amorim *et al.*, 2013 Expression-based and co-localization detection of arabinogalactan protein 6 and arabinogalactan protein 11 interactors in Arabidopsis pollen and pollen tubes. BMC Plant Biol. 13: 7.

Cowan, J. A., 2002 Structural and catalytic chemistry of magnesium-dependent enzymes. BioMetals 15: 225–235.

Czarnecki, O., B. Hedtke, M. Melzer, M. Rothbart, A. Richter *et al.*, 2011 An Arabidopsis GluTR binding protein mediates spatial separation of 5-aminolevulinic acid synthesis in chloroplasts. Plant Cell 23: 4476–91.

Ding, Y., W. Luo, and G. Xu, 2006 Characterisation of magnesium nutrition and interaction of magnesium and potassium in rice. Ann. Appl. Biol. 149: 111–123.

Filiault, D. L., and J. N. Maloof, 2012 A Genome-Wide Association Study Identifies Variants Underlying the Arabidopsis thaliana Shade Avoidance Response. PLoS Genet. 8: e1002589.

Fitz Gerald, J. N., 2005 Identification of Quantitative Trait Loci That Regulate Arabidopsis Root System Size and Plasticity. Genetics 172: 485–498.

Fournier-Level, a., A. Korte, M. D. Cooper, M. Nordborg, J. Schmitt *et al.*, 2011 A Map of Local Adaptation in Arabidopsis thaliana. Science 334: 86–89.

Fulcher, N., A. Teubenbacher, E. Kerdaffrec, A. Farlow, M. Nordborg *et al.*, 2015 Genetic Architecture of Natural Variation of Telomere Length in Arabidopsis thaliana. Genetics 199: 625–635.

Greco, M., A. Chiappetta, L. Bruno, and M. B. Bitonti, 2012 In Posidonia oceanica cadmium induces changes in DNA methylation and chromatin patterning. J. Exp. Bot. 63: 695–709.

Gruber, B. D., R. F. H. Giehl, S. Friedel, and N. von Wiren, 2013 Plasticity of the Arabidopsis Root System under Nutrient Deficiencies. Plant Physiol. 163: 161–179.

Hariadi, Y., and S. Shabala, 2004 Screening broad beans (Vicia faba) for magnesium deficiency. II. Photosynthetic performance and leaf bioelectrical responses. Funct. Plant Biol. 31: 539–549.

Hermans, C., G. N. Johnson, R. J. Strasser, and N. Verbruggen, 2004 Physiological characterisation of magnesium deficiency in sugar beet: Acclimation to low magnesium differentially affects photosystems I and II. Planta 220: 344–355.

Hermans, C., M. Vuylsteke, F. Coppens, A. Craciun, D. Inzé *et al.*, 2010 Early transcriptomic changes induced by magnesium deficiency in Arabidopsis thaliana reveal the alteration of circadian clock gene expression in roots and the triggering of abscisic acid-responsive genes. New Phytol. 187: 119–131.

Hermans, C., M. Vuylsteke, F. Coppens, S. M. Cristescu, F. J. M. Harren *et al.*, 2010 Systems analysis of the responses to long-term magnesium deficiency and restoration in Arabidopsis thaliana. New Phytol. 187: 132–44.

Hoagland, D., and D. Arnon, 1950 The water-culture method for growing plants without soil. Calif Agric Exp Stn Circ 347: 357–359.

Hörtensteiner, S., 2009 Stay-green regulates chlorophyll and chlorophyll-binding protein degradation during senescence. Trends Plant Sci. 14: 155–162.

Horton, M. W., A. M. Hancock, Y. S. Huang, C. Toomajian, S. Atwell *et al.*, 2012 Genome-wide patterns of genetic variation in worldwide Arabidopsis thaliana accessions from the RegMap panel. Nat. Genet. 44: 212–216.

Igamberdiev, A. U., and L. A. Kleczkowski, 2001 Implications of adenylate kinase-governed equilibrium of adenylates on contents of free magnesium in plant cells and compartments. Biochem. J. 360: 225–231.

Karley, A. J., and P. J. White, 2009 Moving cationic minerals to edible tissues: potassium, magnesium,

calcium. Curr. Opin. Plant Biol. 12: 291–298.

Kautz, G., M. Zimmer, P. Zach, J. Kulfan, and W. Topp, 2001 Suppression of soil microorganisms by emissions of a magnesite plant in the Slovak Republic. Water. Air. Soil Pollut. 125: 121–132.

Kellermeier, F., F. Chardon, and A. Amtmann, 2013 Natural Variation of Arabidopsis Root Architecture Reveals Complementing Adaptive Strategies to Potassium Starvation. Plant Physiol. 161: 1421–1432.

Lanquar, V., D. Loque, F. Hormann, L. Yuan, A. Bohner *et al.*, 2009 Feedback Inhibition of Ammonium Uptake by a Phospho-Dependent Allosteric Mechanism in Arabidopsis. Plant Cell 21: 3610–3622.

Li, Y., Y. Huang, J. Bergelson, M. Nordborg, and J. O. Borevitz, 2010 Association mapping of local climate-sensitive quantitative trait loci in Arabidopsis thaliana. Proc. Natl. Acad. Sci. U. S. A. 107: 21199–21204.

Lilley, R. M., K. Holborow, and D. A. Walker, 1974 Magnesium activation of photosynthetic CO2-fixation in a reconstituted chloroplast system . New Phytol. 73: 657–662.

Loudet, O., V. Gaudon, A. Trubuil, and F. Daniel-Vedele, 2005 Quantitative trait loci controlling root growth and architecture in Arabidopsis thaliana confirmed by heterogeneous inbred family. Theor. Appl. Genet. 110: 742–753.

Maguire, M. E., and J. a. Cowan, 2002 Magnesium chemistry and biochemistry. BioMetals 15: 203–210.

Marschner, H., E. a Kirkby, and I. Cakmak, 1996 Effect of mineral nutritional status on shoot-root partitioning of photoassimilates and cycling of mineral nutrients. J. Exp. Bot. 47 Spec No: 1255–1263.

Mikkelsen, R., 2010 Soil and Fertilizer Magnesium. Better Crop. 94: 26–28.

Mouchel, C. F., G. C. Briggs, and C. S. Hardtke, 2004 Natural genetic variation in Arabidopsis identifies BREVIS RADIX, a novel regulator of cell proliferation and elongation in the root. Genes Dev. 18: 700–714.

Niu, Y., G. Jin, X. Li, C. Tang, Y. Zhang *et al.*, 2015 Phosphorus and magnesium interactively modulate the elongation and directional growth of primary roots in Arabidopsis thaliana (L.) Heynh. J. Exp. Bot. 66: 3841–3854.

Niu YF, Chai RS, Liu LJ, Jin GL, Liu M, Tang CX, Z. Y., 2014 Magnesium availability regulates the development of root hairs in Arabidopsis thaliana (L.) Heynh. Plant Cell Env. 37: 2795–2813.

Pyle, A. M., 2002 Metal ions in the structure and function of RNA. J. Biol. Inorg. Chem. 7: 679–90.

Robinson, G. K., 1991 That BLUP is a good thing : the estimation of random effects. Stat. Sci. 6: 15–32.

Rosas, U., A. Cibrian-jaramillo, J. A. Banta, M. L. Gifford, A. H. Fan *et al.*, 2015 Correction for Rosas et al., Integration of responses within and across Arabidopsis natural accessions uncovers loci controlling root

systems architecture. Proc. Natl. Acad. Sci. U. S. A. 112: E2555–E2555.

Schulz, E., T. Tohge, E. Zuther, A. R. Fernie, and D. K. Hincha, 2015 Natural variation in flavonol and anthocyanin metabolism during cold acclimation in A rabidopsis thaliana accessions. Plant. Cell Environ. 38: 1658–1672.

Sergeeva, L. I., J. J. B. Keurentjes, L. Bentsink, J. Vonk, L. H. W. van der Plas *et al.*, 2006 Vacuolar invertase regulates elongation of Arabidopsis thaliana roots as revealed by QTL and mutant analysis. Proc. Natl. Acad. Sci. U. S. A. 103: 2994–2999.

Stein, R. J., and B. M. Waters, 2012 Use of natural variation reveals core genes in the transcriptome of iron-deficient Arabidopsis thaliana roots. J. Exp. Bot. 63: 1039–1055.

Swaminathan, R., 2000 Disorders of magnesium metabolism. CPD Bull. Clin. Biochem. 2: 3–12.

Verslues, P. E., J. R. Lasky, T. E. Juenger, T.-W. Liu, and M. N. Kumar, 2014 Genome-Wide Association Mapping Combined with Reverse Genetics Identifies New Effectors of Low Water Potential-Induced Proline Accumulation in Arabidopsis. Plant Physiol. 164: 144–159.

Visscher, A. M., A.-L. Paul, M. Kirst, C. L. Guy, A. C. Schuerger *et al.*, 2010 Growth performance and root transcriptome remodeling of Arabidopsis in response to Mars-like levels of magnesium sulfate. PLoS One 5: e12348.

Weber, A. P. M., K. L. Weber, K. Carr, C. Wilkerson, and J. B. Ohlrogge, 2007 Sampling the Arabidopsis Transcriptome with Massively Parallel Pyrosequencing. Plant Physiol. 144: 32–42.

Wilkinson, S.R., Welch, Ross M., Mayland, H.F., Grunes, D. L., 1990 Magnesium in Plants: Uptake, Distribution, Function, and Utilization by Man and Animals. Met. Ions Biol. Syst. 26: 33–56.

Williams, L., and D. E. Salt, 2009 The plant ionome coming into focus. Curr Opin Plant Biol 12: 247–249.

Yang, J., T. Ferreira, A. P. Morris, S. E. Medland, P. A. F. Madden *et al.*, 2012 Conditional and joint multiple-SNP analysis of GWAS summary statistics identifies additional variants influencing complex traits. Nat. Genet. 44: 369–75.

Yang, J., L. Tian, M.-X. Sun, X.-Y. Huang, J. Zhu *et al.*, 2013 AUXIN RESPONSE FACTOR17 Is Essential for Pollen Wall Pattern Formation in Arabidopsis. Plant Physiol. 162: 720–731.
