# Supplementary material for: Reproduction and In-Depth Evaluation of Genome-Wide Association Studies and Genome-Wide Meta-analyses Using Summary Statistics
Source: G3 (Bethesda). 2017 Jan 24;7(3):943–52. doi: 10.1534/g3.116.038877 (PMC5345724; doi:10.1534/g3.116.038877)
Supplement: Supplementary file 2 [file 943FileS2.pdf]

OpenGWAS Figure (supplementary)

By
Yao-Fang Niu, et al

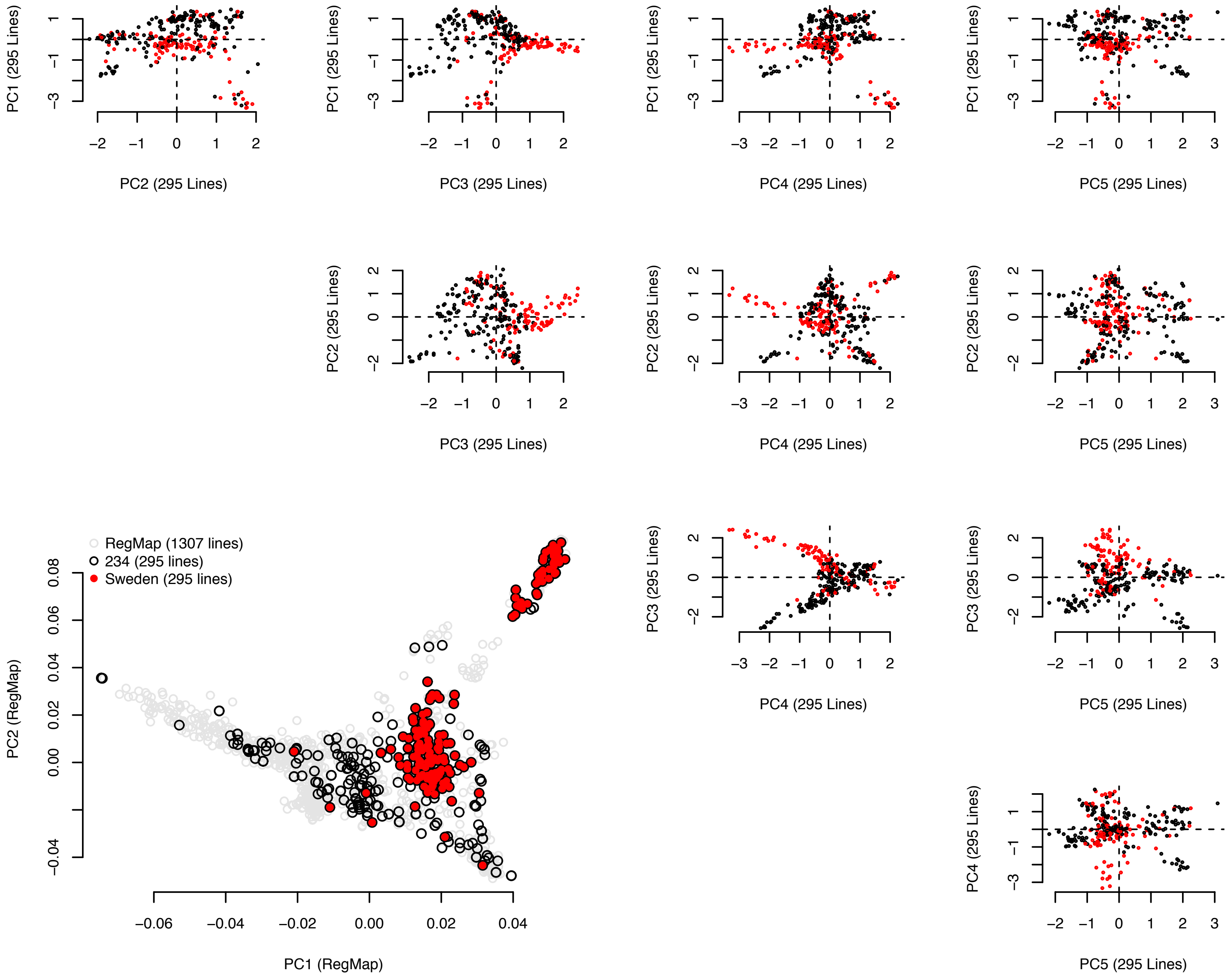

Fig S1A Principal component analysis for 295 Arabidopsis lines. 234 lines are genotyped in 250K chips of the RegMap panel, which includes 1307 lines (Nat Genet, 2012, 44:212-216), and 61 lines are extracted from Arabidopsis 1001 Genomes project. The large plot illustrates the first two eigenvector of the RegMap panel using 156,744 SNPs. The 234 lines are represented in black circles. 178 of the 234 lines are from Sweden (filled in red). The small plots illustrate eigenvectors generated from 295 lines using 157,744 SNPs, and the red points represent the lines from Sweden.

Worldwide distribution for 295 accessions

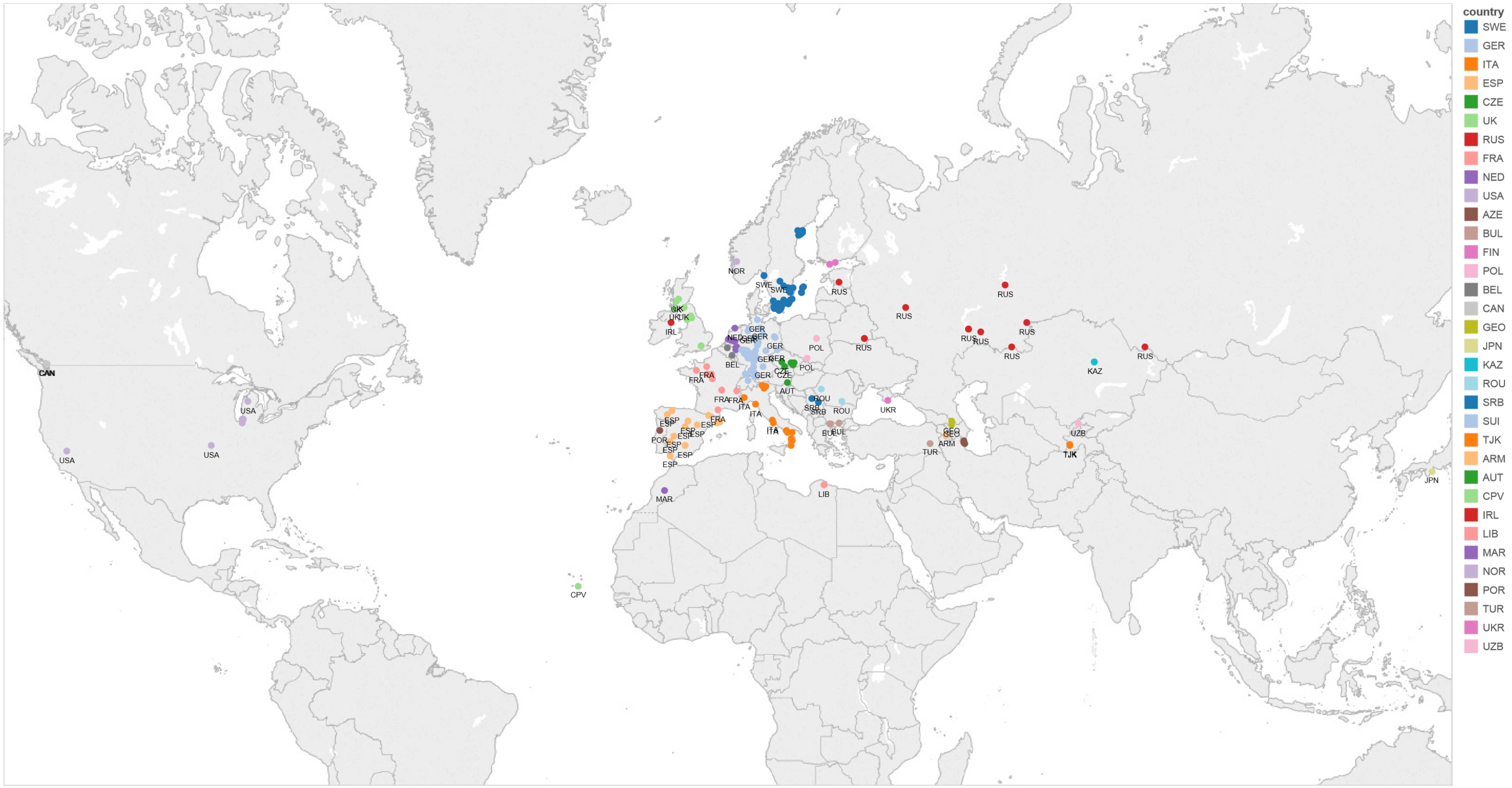

Fig S1B Worldwide diversity of 295 *A. thaliana natural accessions* used in this study according to their reported origins.

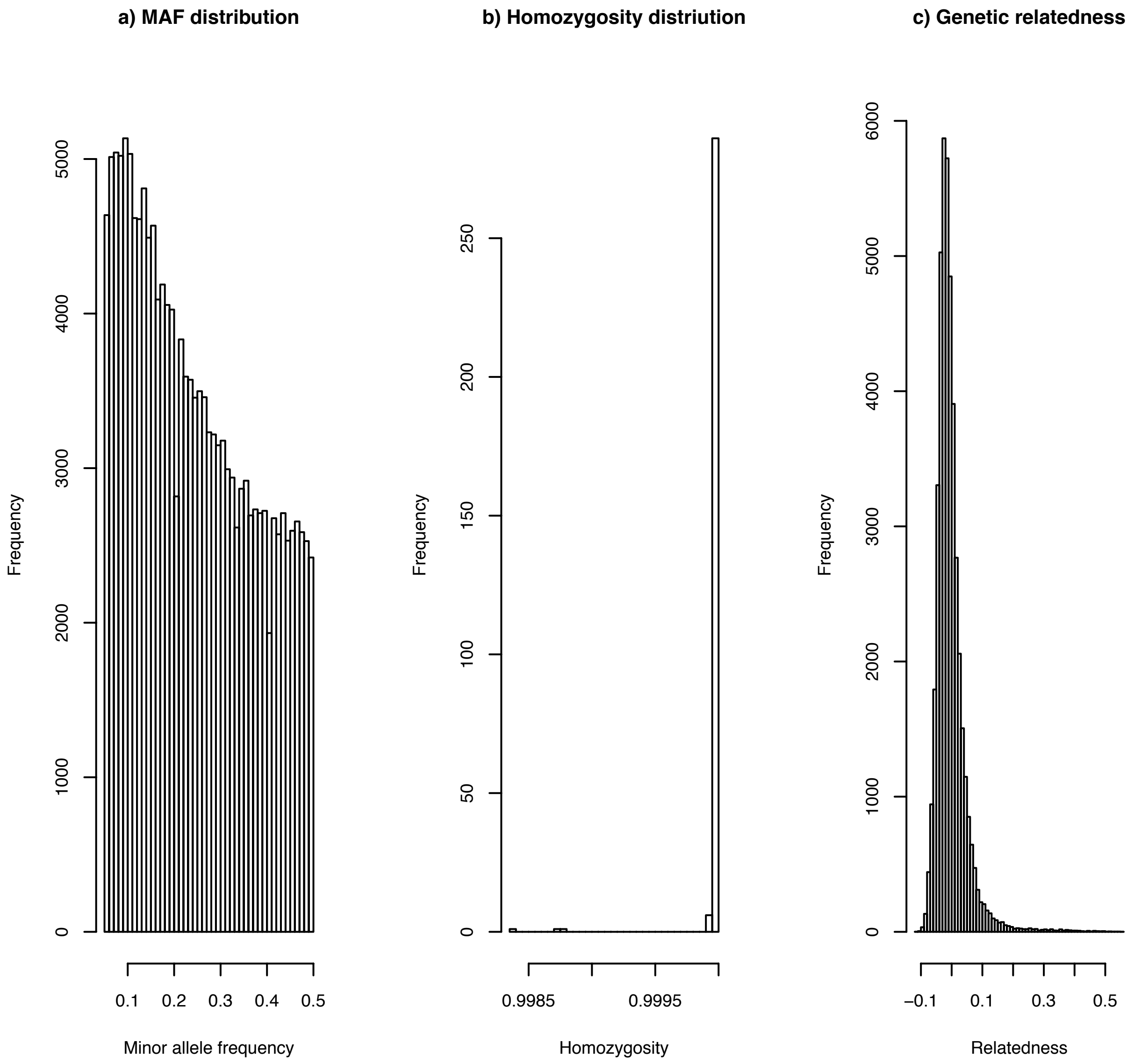

Fig S2 Population parameters for 295 Arabidopsis accessions. a) 156,744 biallelic SNPs having MAF > 0.05 are included in this study. Genotyping rate is 0.998; b) Homozygosity distribution. The mean is 0.9999, consistent to Arabidopsis population, which is a selfing organism; c) Genetic relatedness estimated using 156,744 markers.

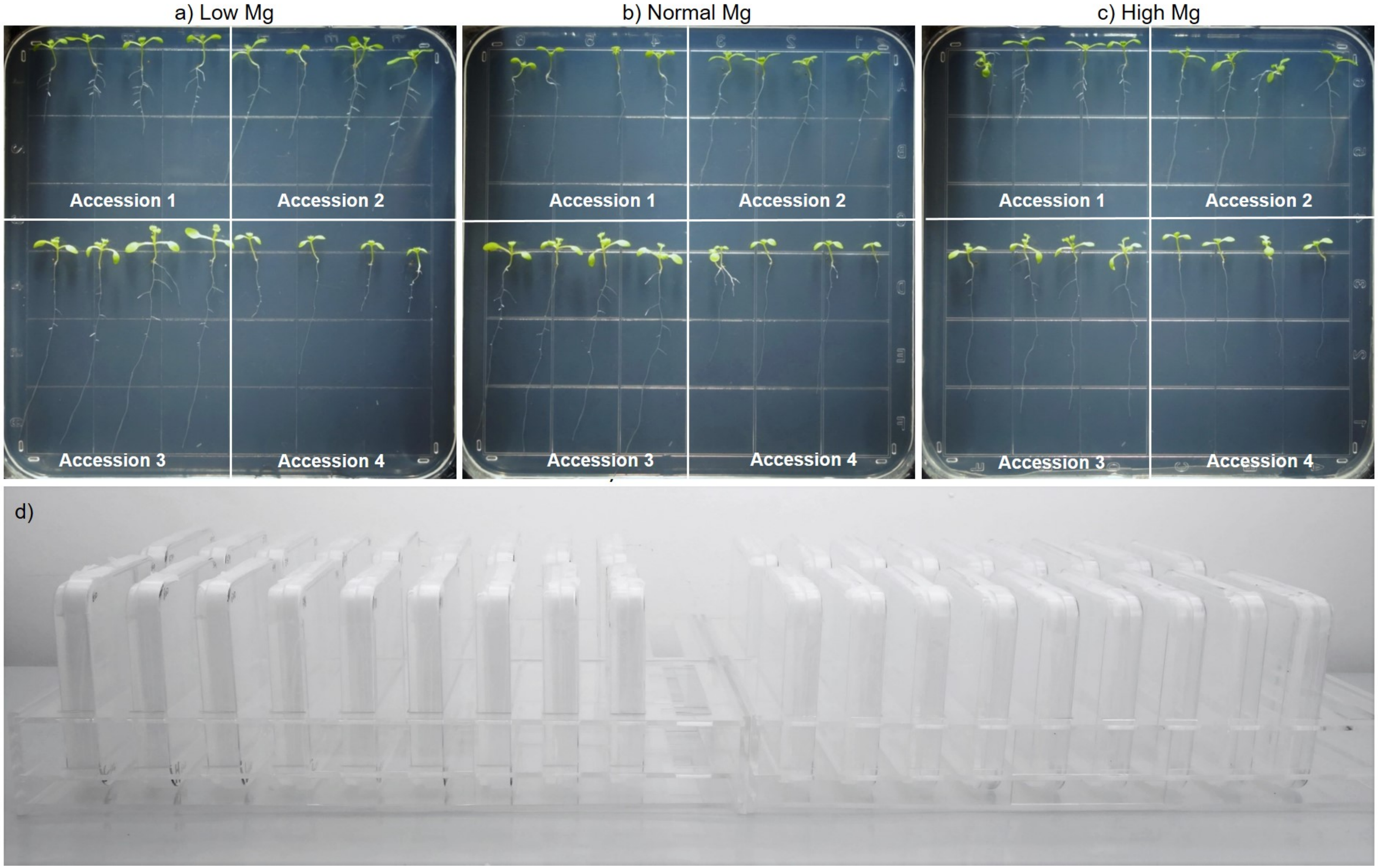

Fig S3 Cultivation for 295 Arabidopsis inbred lines at Zhejiang University, Hangzhou, Zhejiang Province, China. Each line was treated with a) low Mg (1 µM $MgSO_4$), b) normal Mg (1,000 µM $MgSO_4$), and c) high Mg (10,000 µM $MgSO_4$). Each line had four biological replicates, and the median value was used in the analysis. Mg treatments were achieved by altering the concentrations of $MgSO_4$ in the basal medium; d) the racks containing the plates grown vertical at 10 h light/14 h dark photoperiod at constant temperature of 22 ℃, 60 % relative humidity and light intensity of 120 µmol photons m^{-2} s^{-1}.

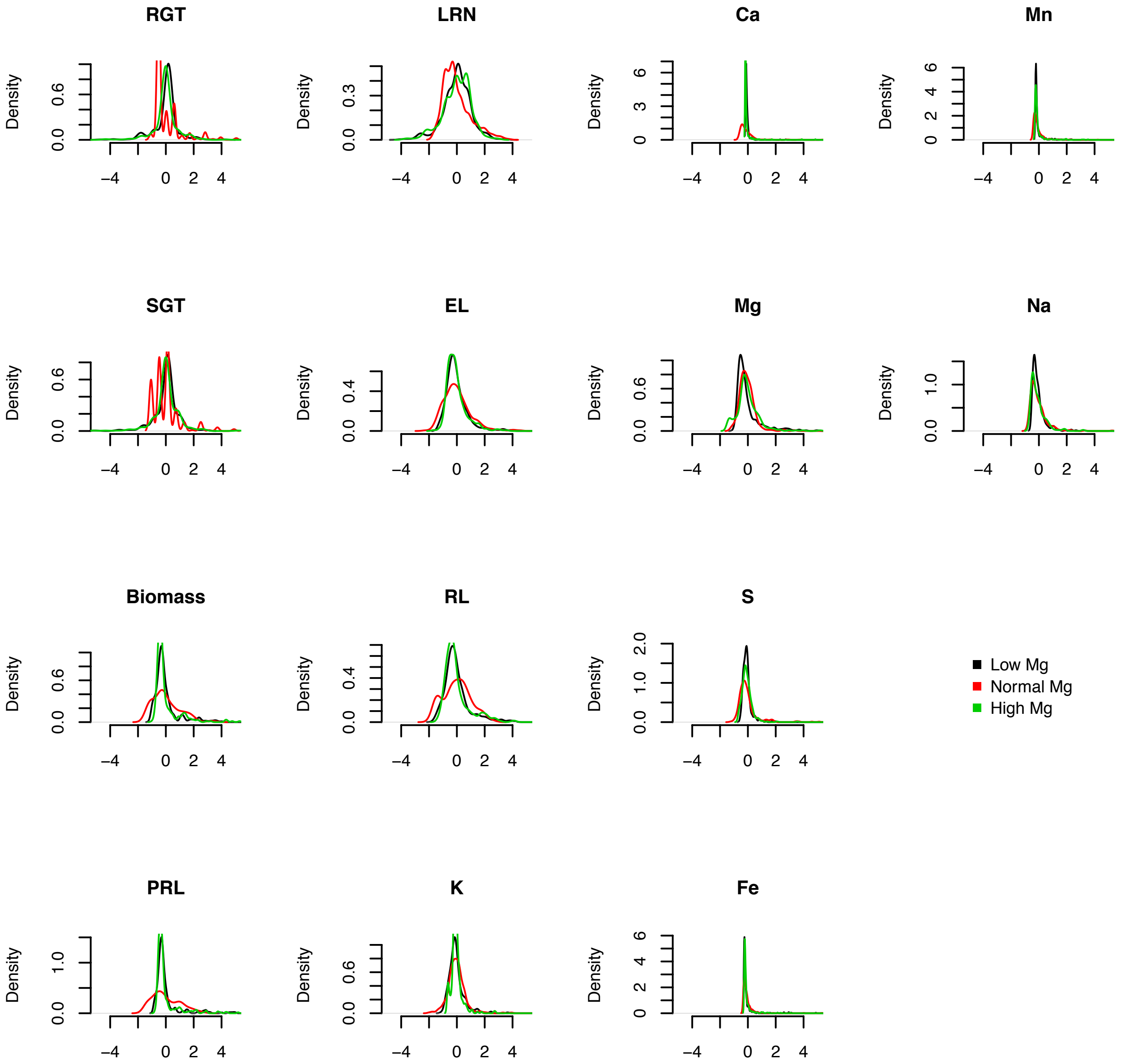

Fig S4 Distribution for 14 traits under three Mg treatments. In each plot the three cures represent the distribution of the trait under three conditions.

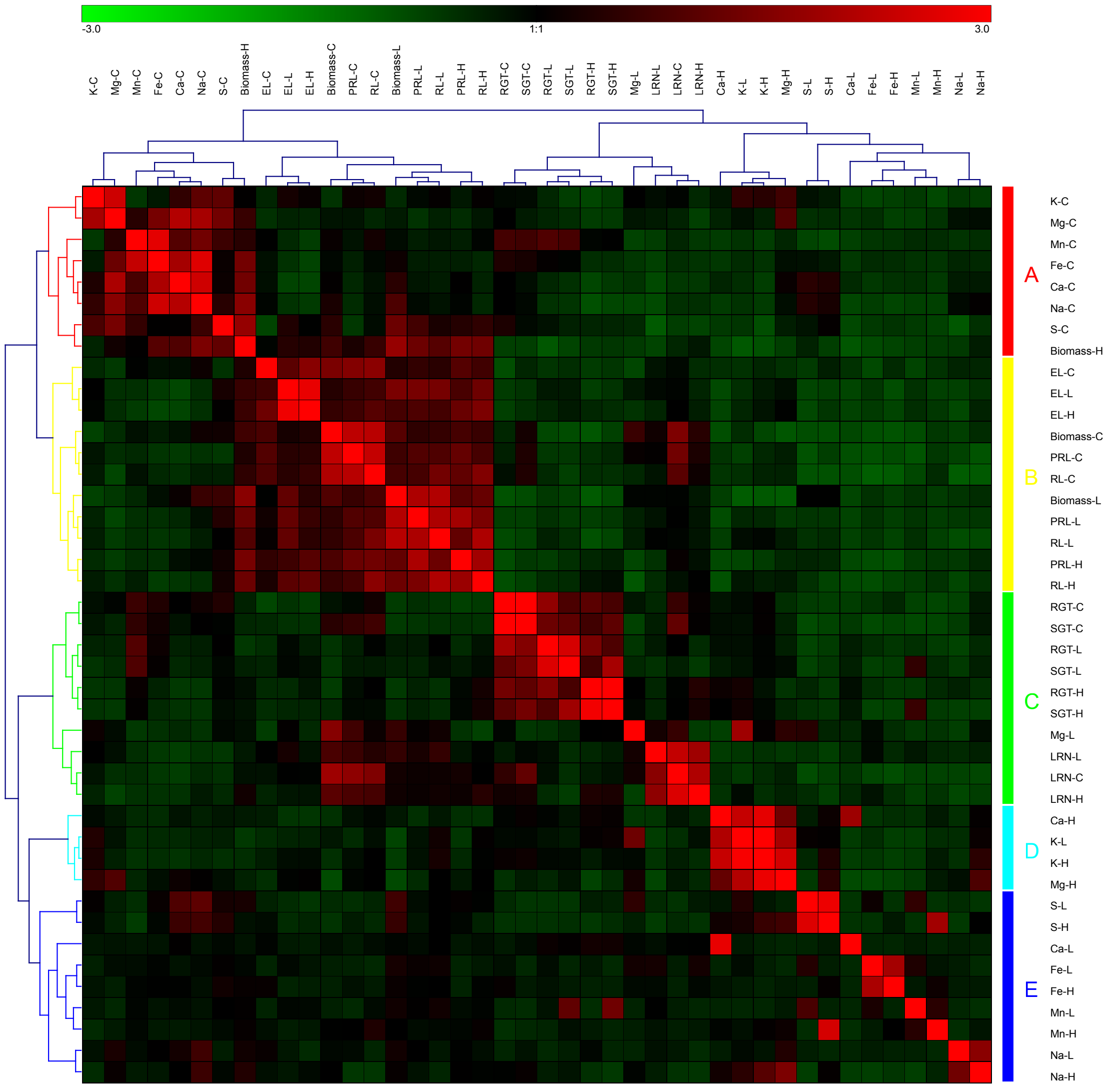

Fig S5 The phenotypic correlation for 42 phenotypes. Heat map represents cluster of the pearson correlation coefficient between each two phenotypes, the pearson's correlation coefficients were normalized (z-score) and represented with blue-red color scheme. These 42 phenotypes were clustered into five classes, denoted as A, B, C, D, and E.

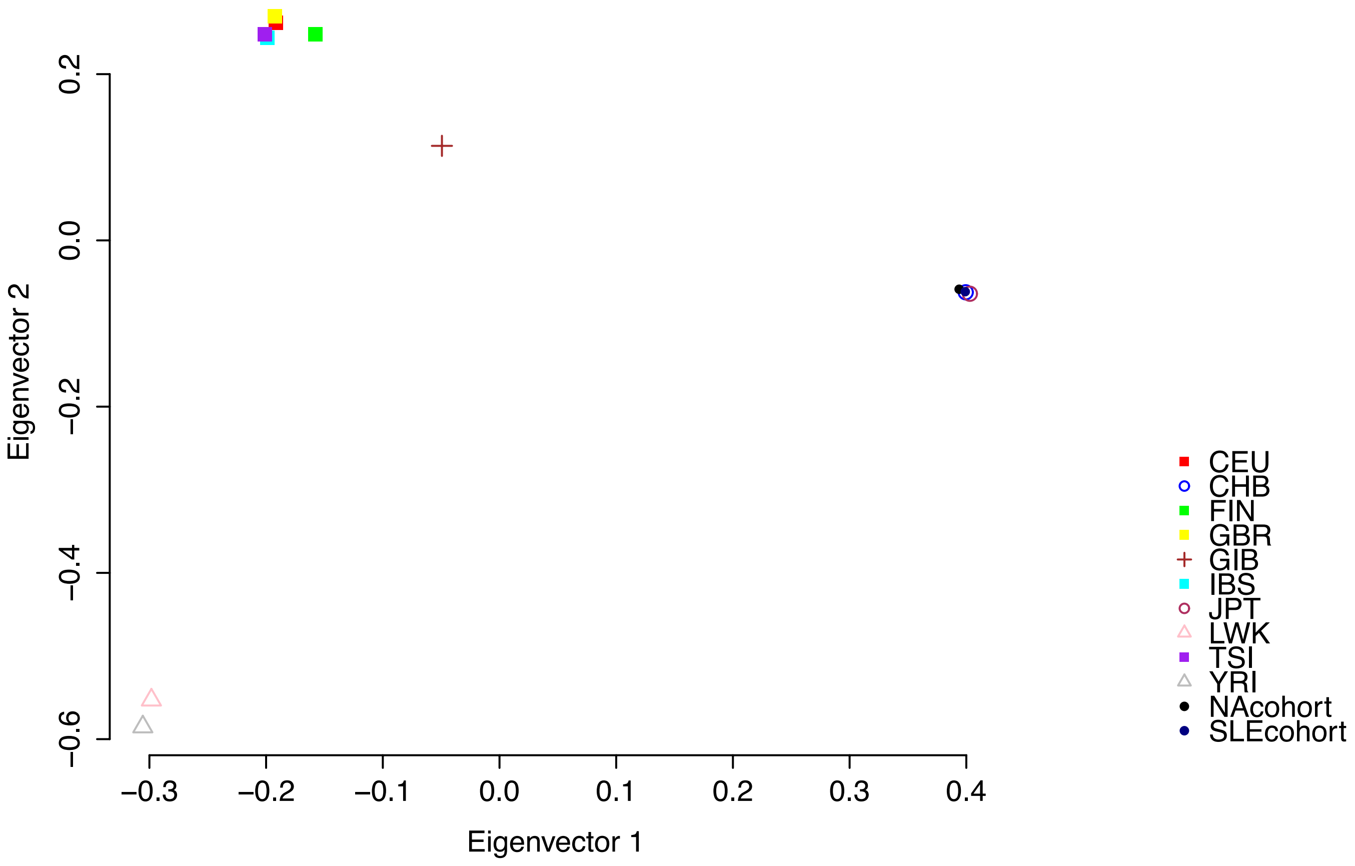

Fig S6 Quality control for NAcohort and SLEcohort using meta-PCA technique. The GWAMA central hub checked the genotype quality of NAcohort and SLEcohort using meta-PCA technique (Chen *et al.* 2017 Euro J Hum Genet), as expected these two Chinese cohorts had been clustered to CHB and JPT cohorts in 1000 Genome Project (The 1000 Genomes Project Consortium 2012)
Cohort codes for 1KG: CEU, CEPH Utah; CHB, Han Chinese in Beijing; FIN, Fins; GBR, British; GIB, Gujarati Indian; IBS, Iberian Population in Spain; JPT, Japanese; LWK, Luhya; TSI, Tuscans in Italy; YRI, Yoruba.

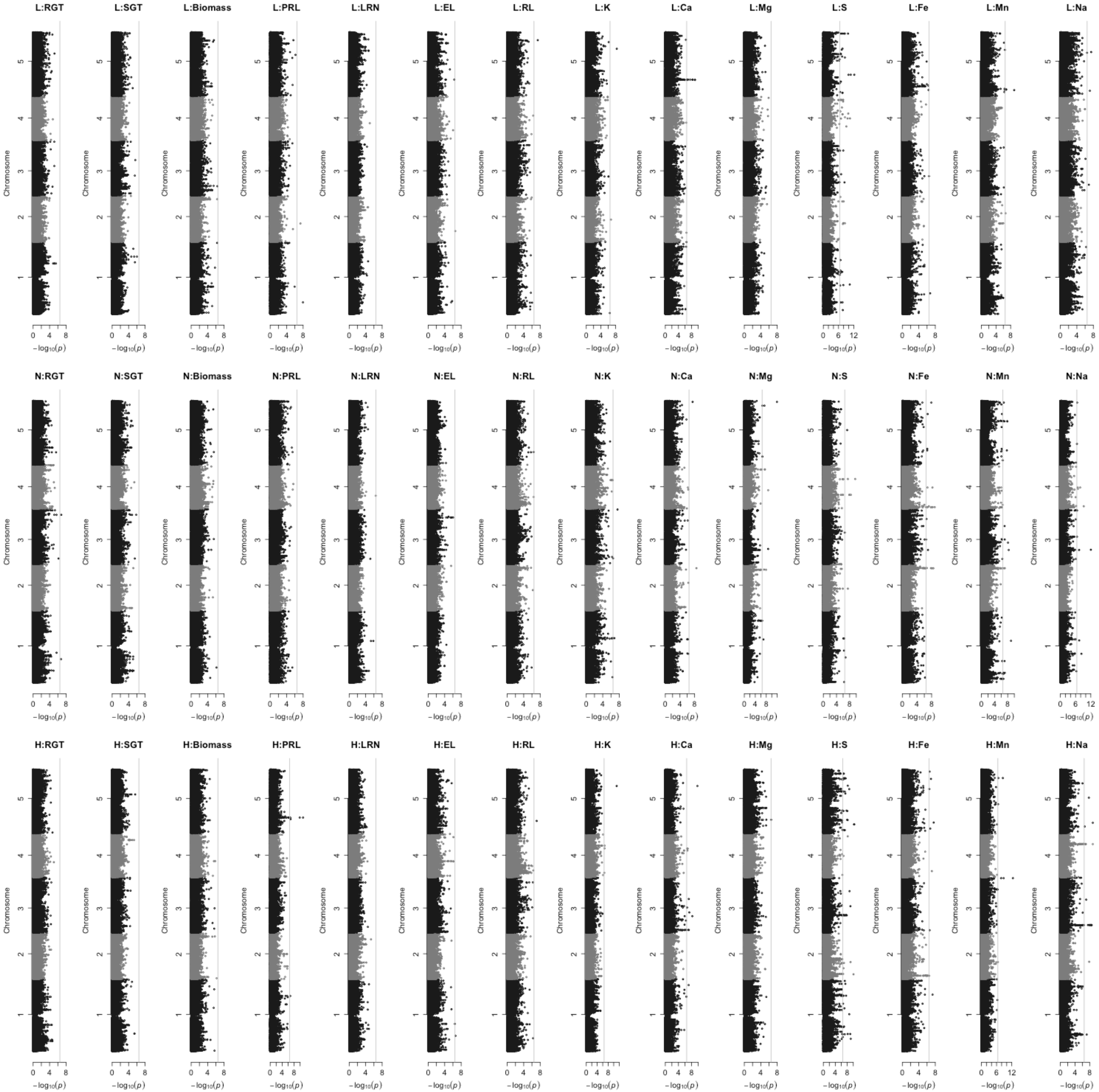

Fig S7 Saturated GWAS for 14 traits under three Mg conditions. The top five eigenvectors were used as covariates. The top row for low Mg, the middle row for normal Mg, and the bottom row for high Mg.

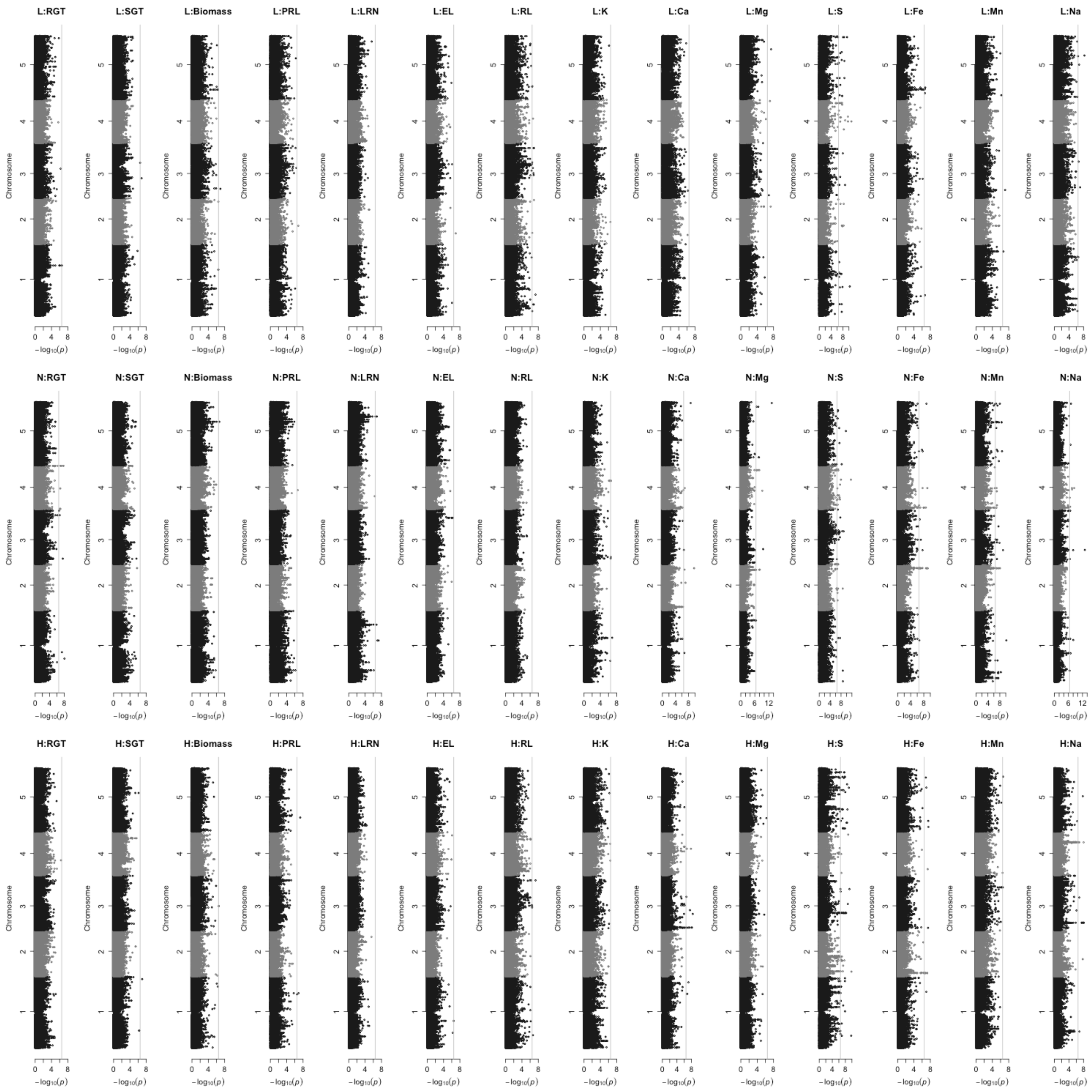

Fig S8 Naïve GWAS for 14 traits under three Mg conditions. The top row for low Mg, the middle row for normal Mg, and the bottom row for high Mg.

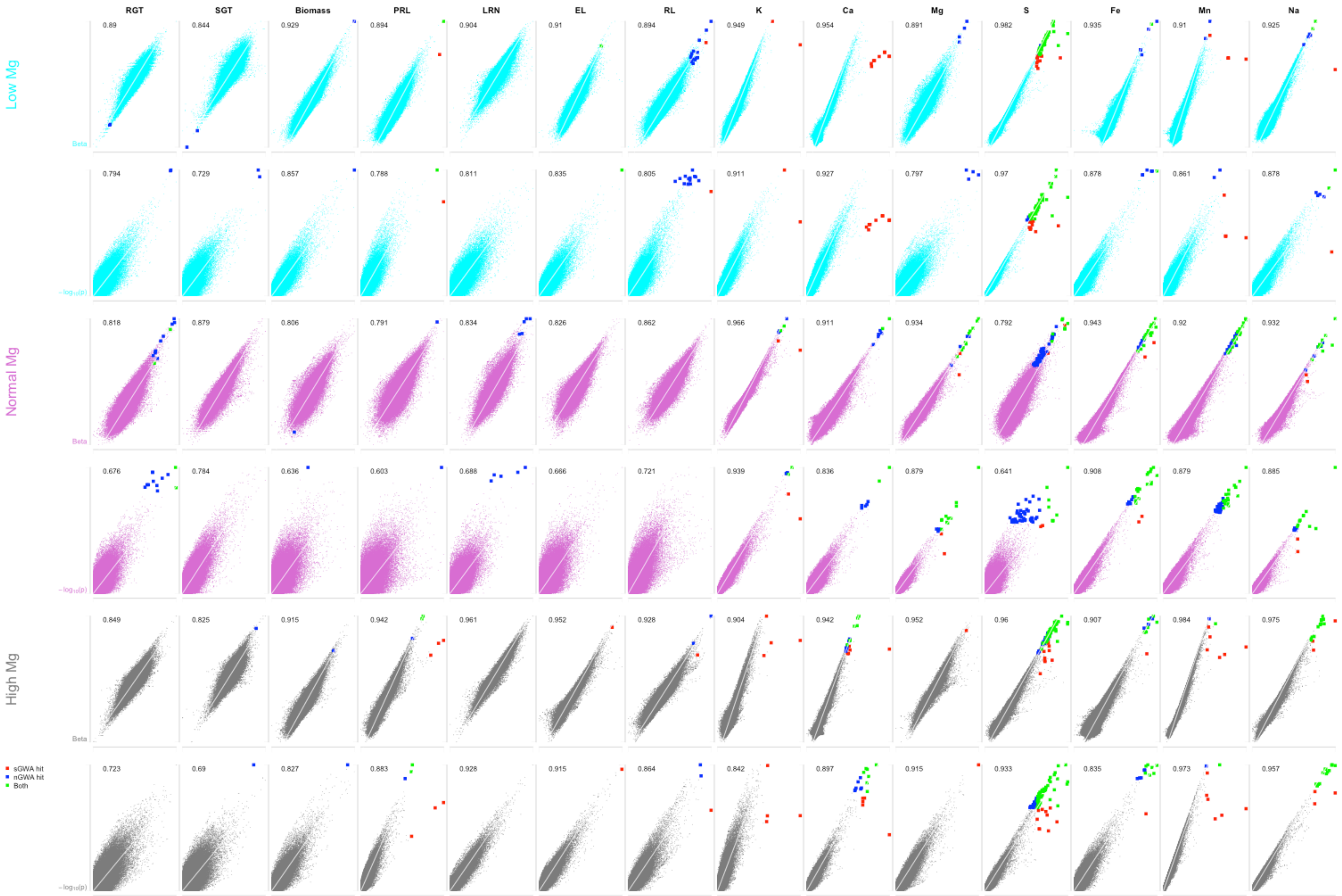

Fig S9 The comparison for the estimated sGWAS and nGWAS statistics (beta and pvalues) for 14 traits under three Mg conditions.
In each panel the x-axis is for the statistics observed in the saturated GWAS models, which were adjusted by the top five eigenvectors, whereas the y-axis for the corresponding statistics observed in the naïve models. The correlations were presented for the statistics. The slope of 1 was in white. The GWAS hits were those had –log10(p) > 6.5. Red ones are sGWAS hits, blue nGWAS hits, and green for hits shared by both models.

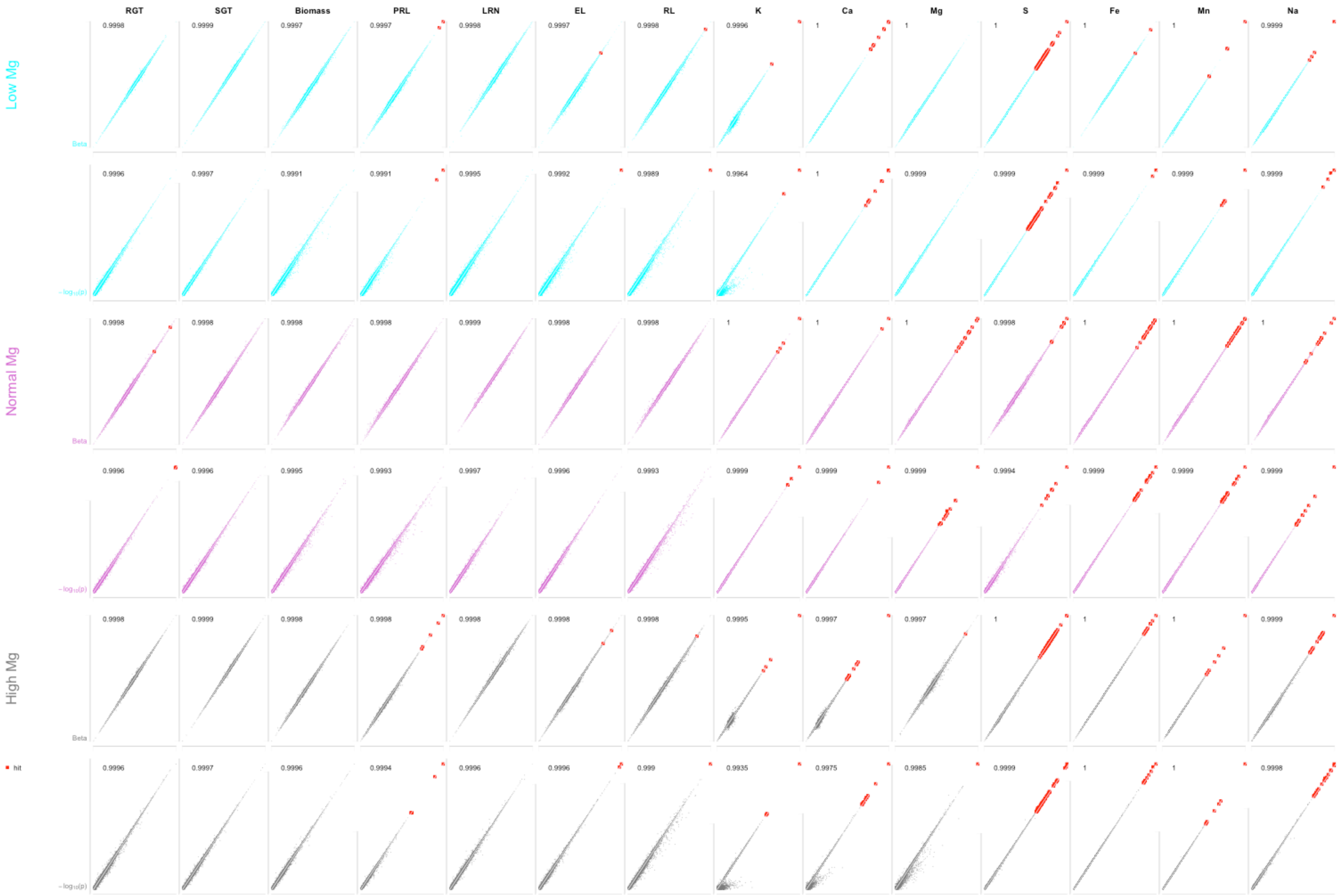

Fig S10 The comparison of beta and –log10(p) estimated from sGWAS that had 5 eigenvectors as covariates and OATH for 14 traits under the three Mg conditions. In each panel, the x-axis represented the either beta or –log10(p) estimated from sGWAS, which was adjusted by the top ten eigenvectors, whereas the y-axis represented the corresponding statistics via OATH. The correlation was represented in each panel, and the GWAS hits, which had OATH –log10(p)>6.5 were in red.
